# Antagonism of miR-328 Increases the Antimicrobial Function of Macrophages and Neutrophils and Rapid Clearance of Non-typeable *Haemophilus Influenzae* (NTHi) from Infected Lung

**DOI:** 10.1371/journal.ppat.1004549

**Published:** 2015-04-20

**Authors:** Hock L. Tay, Gerard E. Kaiko, Maximilian Plank, JingJing Li, Steven Maltby, Ama-Tawiah Essilfie, Andrew Jarnicki, Ming Yang, Joerg Mattes, Philip M. Hansbro, Paul S. Foster

**Affiliations:** 1 Priority Research Centre for Asthma and Respiratory Disease, Department of Microbiology and Immunology, School of Pharmacy and Biomedical Sciences, Faculty of Health and Hunter Medical Research Institute, University of Newcastle, Newcastle, Australia; 2 Priority Research Centre for Asthma and Respiratory Disease, Discipline of Paediatrics and Child Health, School of Medicine and Public Health, Faculty of Health and Hunter Medical Research Institute, University of Newcastle, Newcastle, Australia; NorthWestern University, UNITED STATES

## Abstract

Pathogenic bacterial infections of the lung are life threatening and underpin chronic lung diseases. Current treatments are often ineffective potentially due to increasing antibiotic resistance and impairment of innate immunity by disease processes and steroid therapy. Manipulation miRNA directly regulating anti-microbial machinery of the innate immune system may boost host defence responses. Here we demonstrate that miR-328 is a key element of the host response to pulmonary infection with non-typeable *haemophilus influenzae* and pharmacological inhibition in mouse and human macrophages augments phagocytosis, the production of reactive oxygen species, and microbicidal activity. Moreover, inhibition of miR-328 in respiratory models of infection, steroid-induced immunosuppression, and smoke-induced emphysema enhances bacterial clearance. Thus, miRNA pathways can be targeted in the lung to enhance host defence against a clinically relevant microbial infection and offer a potential new anti-microbial approach for the treatment of respiratory diseases.

## Author Summary

MicroRNAs regulate pathogen recognition pathways by modulating translation. In the immune system, miRNAs have been identified as important regulators of gene expression programs, which regulate differentiation, growth and function of innate and adaptive immune cells. Using miRNA microarray, we demonstrated that lung miRNAs were differentially expressed following non-typeable *Haemophilus Influenzae* (NTHi) infection in mice. To study the role of a specific miRNA in macrophages, we used antagomir (chemically modified single-stranded RNA analogues, complementary to the target miRNA) to block miRNA function. Interestingly, inhibition of microRNA-328 in mouse and human macrophages increases microbicidal activity by amplifying phagocytosis and production of reactive oxygen species. Inhibition of mR-328 in the lung enhanced bacterial clearance in mouse models of immunosuppression and emphysema. Our study provides proof of principle that miRNA pathways can be targeted in the lung and offer a potential new anti-microbial approach for the treatment of respiratory infection.

## Introduction

Pathogenic bacterial infections of the respiratory tract are major causes of morbidity, are difficult to treat and can be life-threatening [1]. They also play a critical role in the pathogenesis of many inflammatory conditions of the lung (e.g. chronic obstructive pulmonary disease (COPD) and cystic fibrosis) and are major causes of acute exacerbations of pre-existing disease [2–4]. Current approaches to the treatment of these diseases and associated exacerbations are often ineffective. A potential explanation is that bacteria are becoming increasingly resistant to antibiotics and effective microbicidal activity requires a robust host defence response, which is impaired in infection-prone patients by underlying disease processes and immunosuppressive therapies (e.g. corticosteroids) [5–9]. A possible new treatment approach is to develop ways of boosting the innate host response, which bacteria cannot easily circumvent.

Recently, important roles for microRNA (miRNA) in regulating innate host defence responses and acquired immunity have been identified [10,11]. In particular, miRNA expression is intimately linked to activation of pathogen recognition pathways (e.g. Toll-Like Receptors (TLR)) that sense invading pathogen and promote immune cell recruitment which in turn leads to elimination of infectious agents. MiRNAs are small non-coding RNAs of approximately 22 nucleotides in length and individual miRNA have the capacity to bind to a multitude of mRNA molecules in a sequence specific manner to inhibit their translation [12]. Thus, a single or set of miRNAs has the potential to control an entire cellular pathway and its related networks. Key examples of miRNAs that are known to be activated by pathogen associated molecular patterns (PAMPs) include miR-9 [13], miR-146 [14] and miR-155 [14], which are important in regulating inflammatory pathways in macrophages and neutrophils by controlling TLR signaling. In addition, recent studies have demonstrated that miRNAs such as miR-155 [15,16], miR-21 [17], and miR-29 [18] are involved in regulating bacterial infections through the innate immune system.

In search of new anti-microbial therapeutic approaches to treat respiratory infections we used non-typeable *Haemophilus Influenzae* (NTHi) as a model to investigate the roles of miRNA in regulating the innate host immune response to infection. NTHi is a commonly isolated bacterium from patients with chronic lung disease and is often linked to exacerbations of COPD [19,20]. During NTHi infection, macrophages and neutrophils are the key innate immune cells recruited to the lung to combat the bacterium. Activation of these cells induces phagocytosis and cytokine secretion that recruits immune cells and facilitates bacterial clearance [21,22]. Here we demonstrate that down-regulation of miR-328-3p (termed miR-328 hereafter) is a key element of the innate host defence response to NTHi infection, which facilitates bacterial clearance. Moreover, pharmacological inhibition of miR-328 profoundly enhances the clearance of the infection by increasing bacterial uptake by phagocytes, the production of reactive oxygen species (ROS), and microbicidal activity. Notably, inhibition of miR-328 in the lung was effective in amplifying the clearance of infection even in models of corticosteroid-induced immunosuppression and cigarette smoke-induced emphysema. Our studies provide the first proof-of-principle data that miRNA pathways can be manipulated in the lung to enhance host defence against microbial infection, and suggest a potential new anti-microbial approach to the treatment of infection induced respiratory diseases.

## Results

### Characterization of miRNA expression during NTHi lung infection

**Fig 1:**
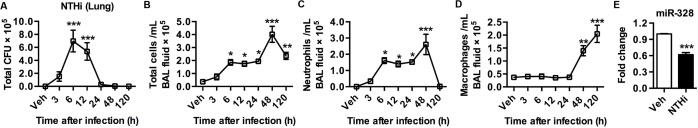
MiR-328 is down-regulated after NTHi infection. (A) NTHi clearance from the lungs. Mice were inoculated i.t. with 5x10^5^ CFU of NTHi. Bacterial load as assessed by bacterial colony counts from lung homogenates. (B) Total cells in BAL fluid following NTHi challenge. (C) Neutrophil and (D) macrophage numbers in BAL fluid determined by differential cell counts. (E) MiR-328 expression levels in airways, validated using Taqman qPCR normalised to sno-202 and expressed as fold change compared to control. Results are expressed as mean ± SEM (n = 5–7 mice per group; * p<0.05, ** p<0.01, *** p<0.001 vs. vehicle control).

We first analyzed the kinetics of bacterial clearance and cellular infiltration into the lungs of NTHi infected BALB/c mice. Mice were inoculated intratracheally (i.t.) with NTHi (5x10^5^ CFU) and infection peaked between 6–12 hours (h) and was cleared within 24 h (Fig 1A). The numbers of total cells (Fig 1B) and neutrophils (Fig 1C) in BAL fluid was significantly increased 6 h post NTHi infection (p.i.). From 24–48 h p.i. total cells (Fig 1B), neutrophils (Fig 1C), and macrophages (Fig 1D) were increased significantly, which coincided with clearance of the infection. To assess the expression and regulation of miRNA during bacterial infection, we performed array analysis on total RNA isolated from the airways of NTHi-infected versus sham-exposed mice 24 h p.i., when cells were actively clearing bacteria. The expression of 15 miRNAs were up-regulated, while 49 were down-regulated >2.5-fold (S1 Fig). We validated the microarray data for several miRNAs using TaqMan PCR and both the pattern of expression and quantitative changes were confirmed. Among these differentially expressed miRNA we selected miR-328, specifically the 3p strand, as a candidate miRNA for further study as its baseline expression was among the highest observed and most significantly down-regulated following infection. Additionally, the roles of miR-328 in immunity to pathogens and in inflammation had not been characterised. We validated the miRNA array data for miR-328 using TaqMan PCR and observed a ~2-fold reduction in expression 24 h after infection (Fig 1E). Interestingly, miR-328 was decreased within 3 h in infected airways and remain decreased over a 48 h period (S2A Fig). Similarly, levels of miR-328 are diminished and remain lower in macrophages isolated from the NTHi infected lung over a similar time period (S2B Fig).

### miR-328 regulates macrophage and neutrophil bacterial phagocytosis

**Fig 2:**
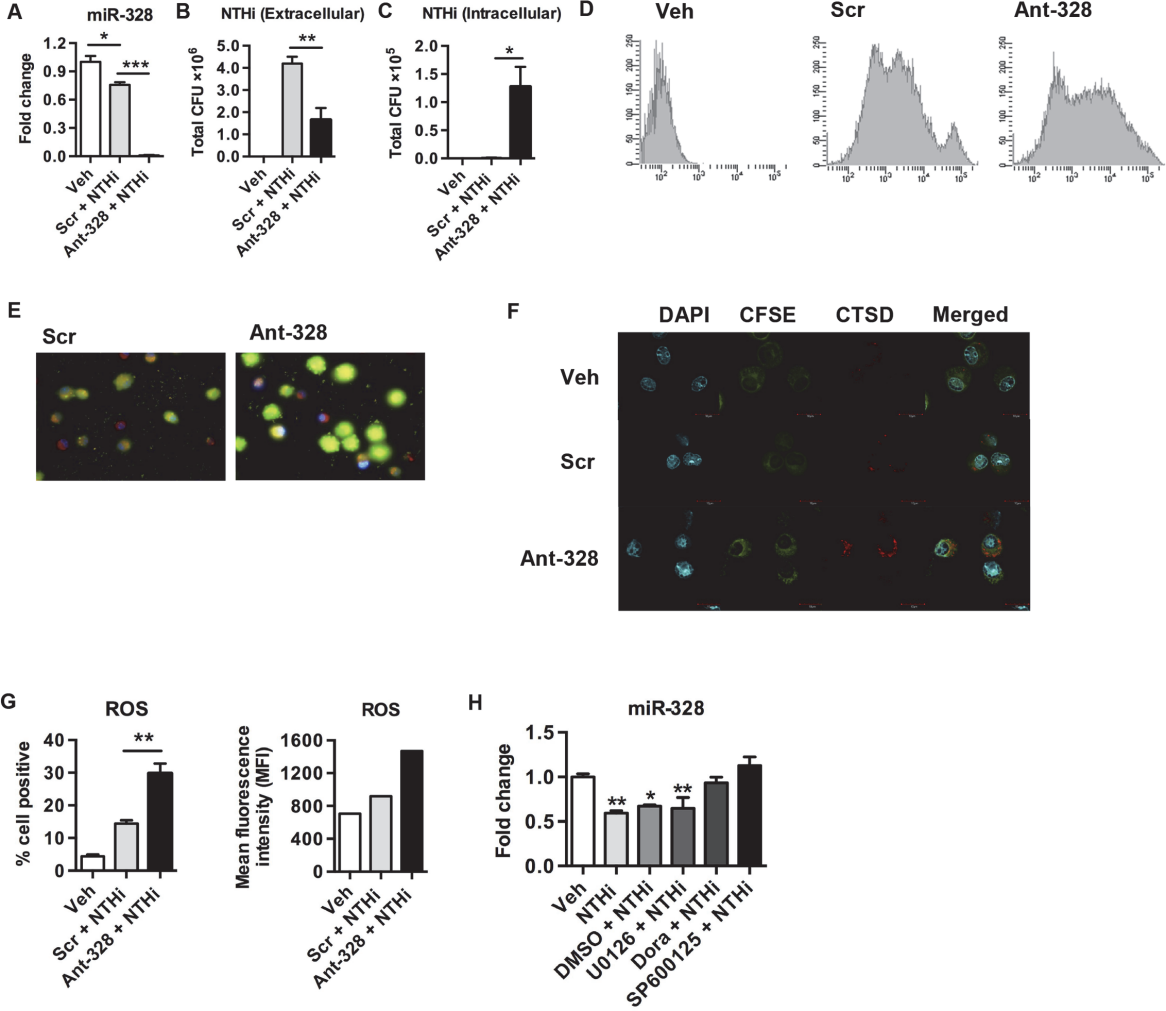
MiR-328 expression is regulated by p38 and JNK MAPK and inhibition increases bacterial clearance *in vitro*. Primary lung macrophages were isolated from the lungs of naive mice and pre-treated with ant-328 for 12 h before infection with NTHi at a MOI 100. Scr was used as a control. (A) Inhibition of miR-328 function with ant-328 validated using Taqman PCR, normalised to sno-202 and expressed as fold change compared to scrambled antagomir-treated controls. (B) Bacterial numbers in culture supernatants measured 8 h post-inoculation, determined by colony counts. (C) Intracellular bacterial counts obtained using gentamicin exclusion assay, whereby gentamicin treatment kills extracellular bacteria prior to cells lysis and remaining intracellular bacteria were plated and counted. (D, E) Phagocytosis assay using heat-killed NTHi. Bacteria were heat-killed and labelled with CFSE, then exposed to macrophages for 1 h. Macrophage intracellular CFSE fluorescence was assessed by (D) flow cytometry and (E) fluorescent microscopy using DAPI (blue) to stain cellular DNA, rhodamine (red) cell protein, and CFSE (green) heat-killed NTHi. (F) Confocal microscopy for cathepsin D expression 8 h post NTHi inoculation. DAPI (blue) cellular DNA, CFSE (green) cell cytoplasm, cathepsin D (red). (G) Dihydroethidium and flow cytometry was used to assess superoxide production in macrophages 8 h following NTHi inoculation. (H) Macrophages were pre-treated with 5 uM doramapimod (Dora), 50 uM SP600125, 50 uM U0126 for 30 min to inhibit p38, JNK, and ERK MAPK activation prior to NTHi infection for 8 h and then miR-328 expression was measured using Taqman qPCR normalised to sno-202 and expressed as fold change compared to sham-inoculated controls. Results are expressed as mean ± SEM (n = 3–4 samples per group; * p<0.05, ** p<0.01, *** p<0.001 as indicated on graphs).

To examine the function of miR-328 in microbial host defence responses in the lung, we isolated the two major innate immune cells that respond to infection and were elevated in the lungs; macrophages and neutrophils. Macrophages were purified from lungs of naïve mice and pre-treated with a miRNA inhibitor (antagomir (ant)) with perfect complementarity to miR-328 (ant-328). This blocked miR-328 function before the cells were infected with NTHi *in vitro*. An antagomir with a scrambled sequence (Scr) was used as a negative control. Antagomir concentrations were titrated such that at the dose used approximately 100% of macrophages (or neutrophils) in the cultures contained cytoplasmic antagomir. Exposure of macrophages to NTHi and Scr resulted in a decrease in the levels of miR-328 by ~25% as assessed by TaqMan qPCR (Fig 2A) (this level was similar to NTHi exposure alone). Administration of ant-328 inhibited expression of miR-328 (Fig 2A). Macrophages treated with ant-328 had a significantly reduced bacterial load in the culture supernatant and increased bacterial uptake compared to the Scr treated controls (Fig 2B and 2C). Similar results were obtained with neutrophils purified from the bone marrow of naïve mice and exposed to the above treatments. Inhibition of miR-328 in neutrophils (S3A Fig) resulted in a 3-fold decrease in bacterial load in the culture supernatant as early as 1 h p.i. and a dramatic increase in bacterial uptake compared to Scr control treatment (S3B–S3C Fig, respectively).

To determine whether the effects of miR-328 inhibition were due to altered phagocytosis of bacteria, or increased permissibility of phagocytes to active infection, we conducted a phagocytosis assay. Macrophages or neutrophils were exposed to heat-killed NTHi (to remove its ability to infect cells) labelled with CFSE and treated with ant-328 (or Scr control) *in vitro* for 1 h, and uptake assessed both by flow cytometry and fluorescence microscopy. Inhibition of miR-328 function in macrophages (Fig 2D and 2E) and neutrophils (S3D Fig) substantially enhanced phagocytosis of heat-killed NTHi (significantly increased levels of intracellular CFSE). The use of trypan blue in these experiments to quench CFSE fluorescence from extracellular bacteria attached to the cell surface confirmed the increased intracellular uptake after ant-328 treatment. To directly demonstrate that ant-328 increased the phagocytosis of bacteria we pre-treated macrophages with both antagomir and cytochalasin D (a potent phagocytosis inhibitor) before exposing the cells to heat-killed CFSE-labelled bacteria. Inhibition of phagocytsosis by cytochalasin D significantly reduced the effect of ant-328 on uptake of bacteria (S4 Fig). The marked effect on bacterial clearance by ant-328 was not related to apoptosis since there were no significant difference in the rate of cell death following antagomir treatment in either macrophages or neutrophils (S5A and S5B Fig). Treatment of macrophages with the mimetic to miR-328 had no effect on bacterial clearance likely because the basal expression level of miR-328 in lung macrophages is already high and so any additive effect becomes masked (S6A and S6B Fig). We next determined how the effects of ant-328 on enhancing phagocytosis may be regulated by examining specific bacterial binding and uptake pathways. We observed that treatment of macrophages with ant-328 significantly increased the expression of multiple bacterial uptake pathways including the LPS binding molecule CD14, the non-opsonic scavenger receptor CD36, and the bacterial adhesion integrin CD11b (S7A and S7C Fig). Collectively, this data suggests that miR-328 regulates bacterial phagocytosis in neutrophils and macrophages at least partly through the control of cell surface bacterial binding proteins.

### miR-328 regulates bacterial killing pathways and its expression is controlled by p38 and JNK signalling

To mechanistically extend our observations, we looked at some important events that occurred following phagocytosis. Using confocal microscopy, we showed that pre-treatment of macrophages with ant-328 increased expression of the lysosomal enzyme, Cathepsin D (increased red fluorescence in cytoplasm), following NTHi infection compared to Scr control treated macrophages (Fig 2F). Activation of the respiratory oxidative burst and the production of ROS is another crucial event in bacterial killing by lysosomes in phagocytes. Thus, we determined whether miR-328 plays a role in activating these killing pathways in macrophages and neutrophils. Following NTHi infection, pre-treatment of macrophages with ant-328 significantly increased both the number of cells producing ROS and the intensity of its production per cell compared to Scr treated controls (Fig 2G). Likewise, the inhibition of miR-328 in neutrophils produced similar results (S3*E* Fig). Importantly, in neutrophils ROS production was increased by ant-328 treatment even in the absence of NTHi, although to a lower extent, suggesting that the effect on these bacterial killing pathways was partly independent of phagocytosis (S8A and S8B Fig). Interestingly, the increased killing was not associated with pro-inflammatory cell activation as the major pro-inflammatory cytokines produced by infection, IL-6 and TNF-α, were not altered by ant-328 treatment of macrophages (S9A and S9B Fig). Thus, miR-328 plays a very specific role in the microbicidal activity of these innate immune cells.

To investigate the signalling pathways involved in the regulation of miR-328 expression, we used specific inhibitors to block the activation of p38, JNK, and ERK MAPK following NTHi infection. Exposure of macrophages to NTHi or vehicle (DMSO) plus NTHi led to a significant 2-fold reduction in miR-328 expression (Fig 2H). However, pre-treatment of macrophages with a p38 inhibitor, doramapimod, or a JNK inhibitor, SP600125, prior to NTHi infection, completely blocked the down-regulation of miR-328 expression (Fig 2H). In contrast, the ERK inhibitor, U0126, had no effect. This suggests that NTHi down regulates miR-328 at least in part through p38 and JNK signalling pathways. We confirmed that NTHi exposure to primary macrophages activates p38 (S10A Fig) and JNK (S10B Fig) but not ERK (S10C Fig) signalling.

We then determined whether other miRNAs that were also identified from the array data (S1 Fig) as having increased or decreased expression in the lungs of mice following NTHi infection, could also play a role in bacterial clearance. Antagomir-mediated inhibition of miR-21-3p and miR-223 (increased expression following bacterial infection), or miR-376c (decreased expression), and miR-21 (control, no change in expression) had no effects on NTHi clearance by neutrophils *in vitro* (S11 Fig). Collectively, these data suggest that NTHi activates the p38 and JNK signalling pathways, which regulates the cellular levels of miR-328. Inhibition of miR-328 with ant-328 suggests that this microRNA regulates phagocytosis and killing of bacteria by macrophages and neutrophils *in vitro*.

### MiR-328 regulates bacterial clearance by macrophages and neutrophils *in vivo*

**Fig 3:**
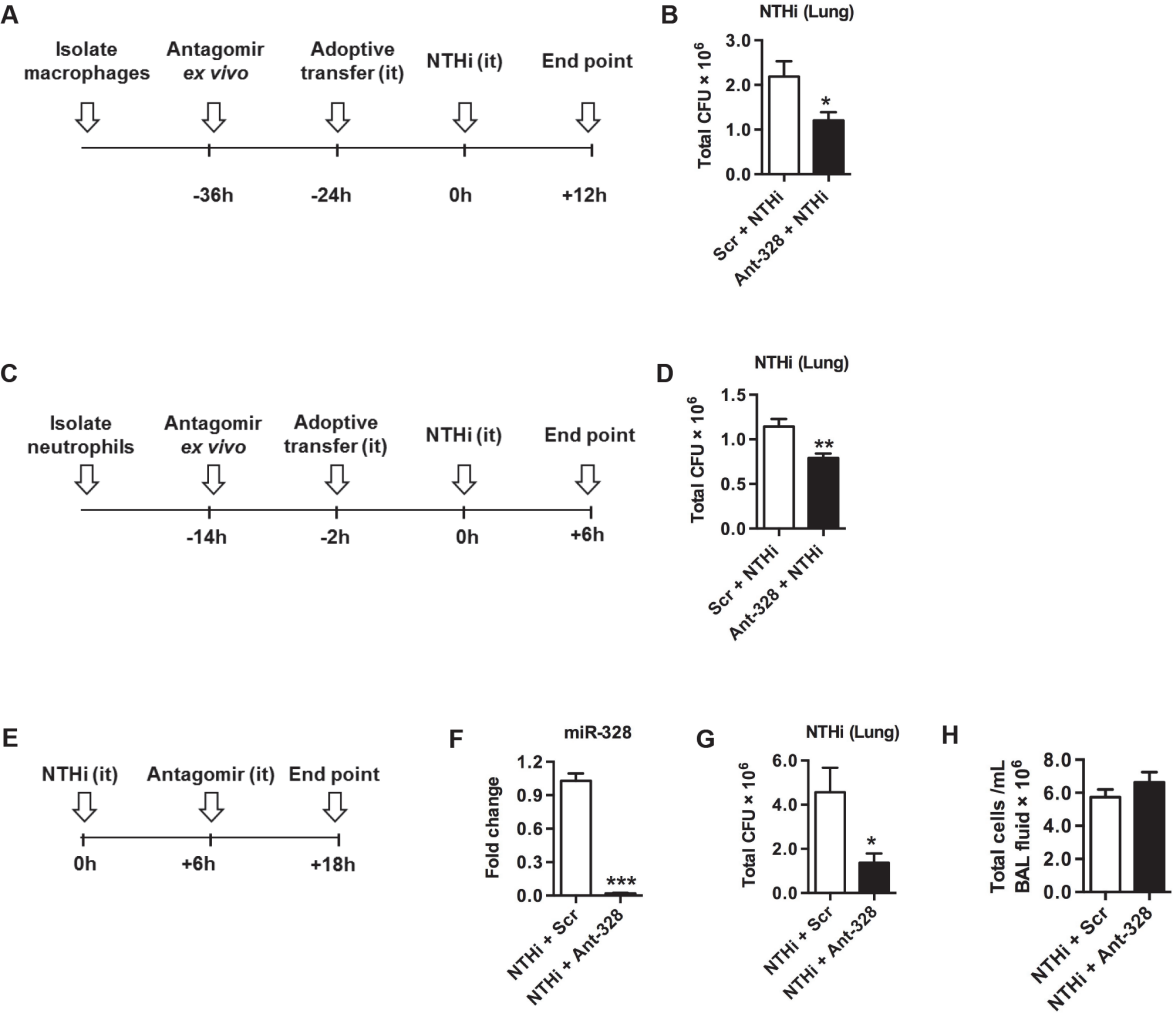
Inhibiting miR-328 improves NTHi clearance in the lungs *in vivo*. MiR-328 was inhibited in (A) macrophages or (C) neutrophils for 12 h *ex vivo*. Then macrophages or neutrophils were labelled with CFSE and adoptively transferred i.t. into naïve mice, which were then infected with NTHi according to the timelines depicted. (E) Mice were inoculated with NTHi and 6 h later ant-328 or scrambled antagomir was administered to the lungs i.t. After 12 h (F) miR-328 expression was determined using Taqman PCR, normalised to sno-202 and expressed as fold change compared to the scrambled antagomir treatment. (B,D,G) Bacterial load in the lungs was measured by plating and counting bacterial colonies from lung homogenates in each model, and (H) BAL fluid was collected, cells isolated by cytospin and stained, and the total cellular infiltrate was assessed. Results are expressed as mean ± SEM (n = 6–8 mice per group; * p<0.05, ** p<0.01, *** p<0.001 vs scrambled antagomir control).

To specifically assess the role of miR-328 in macrophage- and neutrophil-mediated bacterial clearance *in vivo* we conducted adoptive transfer experiments. Macrophages or neutrophils were isolated from naïve mice and then treated with ant-328 or Scr control for 12 h before i.t. transfer into recipient naïve BALB/c mice (Fig 3A and 3C). To monitor the effectiveness of transfer, cells were pre-stained with CFSE. Mice were then inoculated with NTHi. Both CFSE-labelled macrophages (S12B and S12C Fig) and neutrophils (S12E and S12F Fig) entered the lungs and remained there for at least 24 and 2 h, respectively. There were no differences in the numbers of these cells between ant-328- and Scr-treated controls. Total bacterial load in BAL fluid and lung homogenates was measured by plating and colony counting. Importantly, mice that received ant-328 treated macrophages (Fig 3A and 3B) or neutrophils (Fig 3C and 3D) showed significantly improved clearance of NTHi from the lungs compared to Scr control treatment. However, there were no statistical differences in total inflammatory cell infiltrates in BAL fluid (S13A and S13F Fig). Mice that received ant-328 treated macrophages had similar total number of macrophages (S13B Fig) but a significantly reduced number of neutrophils (S13C Fig). It is likely that the reduction in neutrophil numbers is due to increased clearance of bacteria by ant-328 which leads to reduction in the early inflammatory events that promote neutrophil influx. The adoptive transfer of macrophages is likely to promote the removal of apoptotic neutrophils as well. Mice that received ant-328 treated neutrophils had no difference in macrophages (S13G Fig) or neutrophils numbers (S13H Fig). The concentrations of pro-inflammatory cytokines, IL-6 and TNF-α, in BAL fluid were equivalent in all experiments (S13D–S13E and S13I–S13J Fig). These data suggest that the effect of ant-328 on bacterial clearance was mediated directly by phagocytes and not by increased inflammatory cell recruitment or cytokine production.

### Inhibition of miR-328 in the lungs promotes bacterial clearance

We next assessed whether inhibition of miR-328 improved NTHi clearance *in vivo*, which would indicate whether the use of ant-328 would be a potential therapeutic option for respiratory bacterial infection. Naïve BALB/c mice were inoculated with NTHi, and 6 h later were treated i.t. with ant-328 or Scr control (Fig 3E). Inhibition of miR-328 function by ant-328 was confirmed during infection (Fig 3F). NTHi clearance from the lungs was significantly enhanced (4-fold) by ant-328 treatment compared to controls (Fig 3G). Again total (Fig 3H) and neutrophils infiltration (S13L Fig) in the BAL and the levels of the pro-inflammatory cytokines, IL-6 and TNF-α, were not significantly altered (S13M–S13N Fig). In contrast, there was a small but significant increase in the number of macrophages recruited to the lungs by ant-328 treatment following NTHi infection (S13K Fig). We then tested whether the small changes in inflammatory cell numbers in the BAL in some experiments was due to the direct effect of ant-328, or indirectly due to a secondary effect induced by alterations in NTHi clearance after ant-328 treatment. In order to assess this we treated naïve mice with Scr or ant-328 and measured inflammatory cells and cytokines in the BAL. We observed that there was no effect of ant-328 on BAL cell recruitment or pro-inflammatory cytokine production in non-infected mice (S14A–S14D Fig).

### Inhibition of miR-328 overcomes corticosteroid-induced immune suppression to clear bacteria

The mainstay treatment of many respiratory diseases is the prolonged use of corticosteroids, however this leads to immunosuppression and increased risk of hospitalisation for pneumonia, especially in COPD patients [23]. This has been shown in rodent and human studies to result from the suppression of phagocytosis of bacteria by macrophages [24–26].

**Fig 4:**
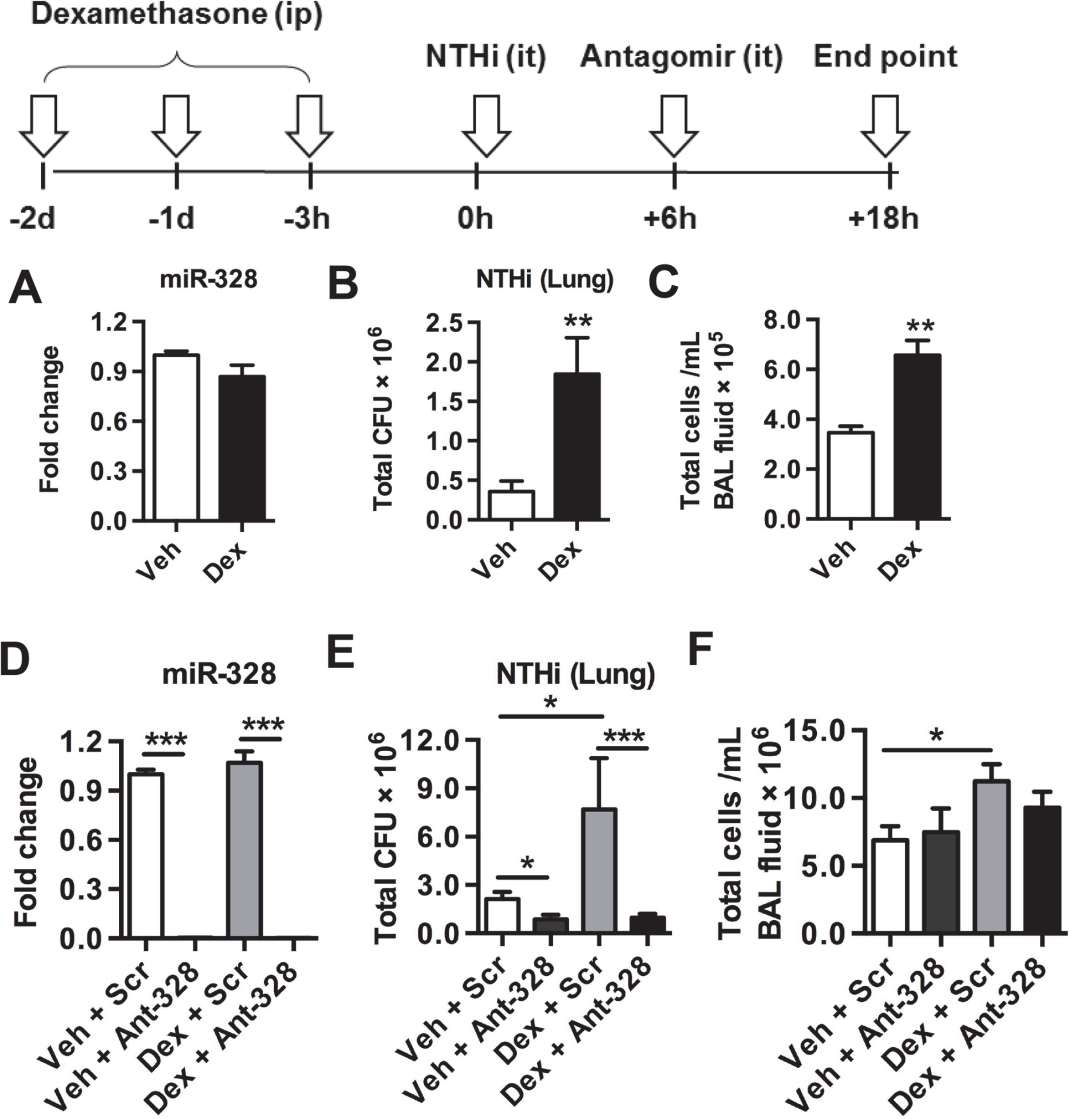
Inhibiting miR-328 enhances bacterial clearance in dexamethasone-mediated immune suppressed mice. (A-C) Mice were treated with vehicle or dexamethasone (i.p.) for 3 consecutive days before being challenged with NTHi (i.t.) for 18hr. (D-F) Mice were treated with antagomir or scrambled control 6 h post inoculation. (A, D) MiR-328 expression was determined using Taqman PCR, normalised to sno-202 and expressed as fold change compared to vehicle and scrambled control groups. (B, E) bacterial load in the lungs was measured by plating and colony counting lung homogenates, and (C, F) BAL fluid was collected, cells isolated by cytospin and stained, and the total cellular infiltrate was assessed. Results are expressed as mean ± SEM (n = 7–12 mice per group; * p<0.05, ** p<0.01, *** p<0.001 as indicated on the graphs).

We next determined whether miR-328 was involved in dexamethasone-mediated suppression of immunity and bacterial clearance, and if inhibition of this miRNA could enhance bacterial clearance in these immune suppressed mice. Mice were administered dexamethasone i.p. daily for 3 days and were then inoculated with NTHi (scheme Fig 4). Dexamethasone treatment did not alter miR-328 expression, suggesting that this miRNA was not directly involved in mediating the effects of corticosteroids (Fig 4A). As expected, dexamethasone pre-treated mice inoculated with NTHi had substantially increased bacterial load in their lungs during infection (Fig 4B). Interestingly, pre-treatment also increased the inflammatory cell infiltrate in the lungs. This is likely due to the increased bacterial load, which results in a greater proinflammatory response leading to the recruitment of more inflammatory cells (Fig 4C). Ant-328 treatment inhibited miR-328 in both dexamethasone and vehicle-treated mice infected with NTHi to similar levels (Fig 4D). Inhibition of miR-328 in dexamethasone pre-treated mice reversed the increased bacteria load compared to controls (Fig 4E). Importantly, bacterial levels were reduced to below those in non-immune-suppressed mice (vehicle+Scr control), and to equivalent levels compared to non-immune suppressed mice treated with ant-328 (vehicle+ant-328). These data indicate that the inhibition of miR-328 was as effective at clearing bacteria in both immune competent and immune suppressed environments. Again the number of infiltrating inflammatory cells in the BAL was only increased by dexamethasone treatment and there was no difference between Scr control and the ant-328 treated groups (Fig 4F).

### Inhibition of miR-328 promotes bacterial clearance in a model of cigarette smoke-induced emphysema

**Fig 5:**
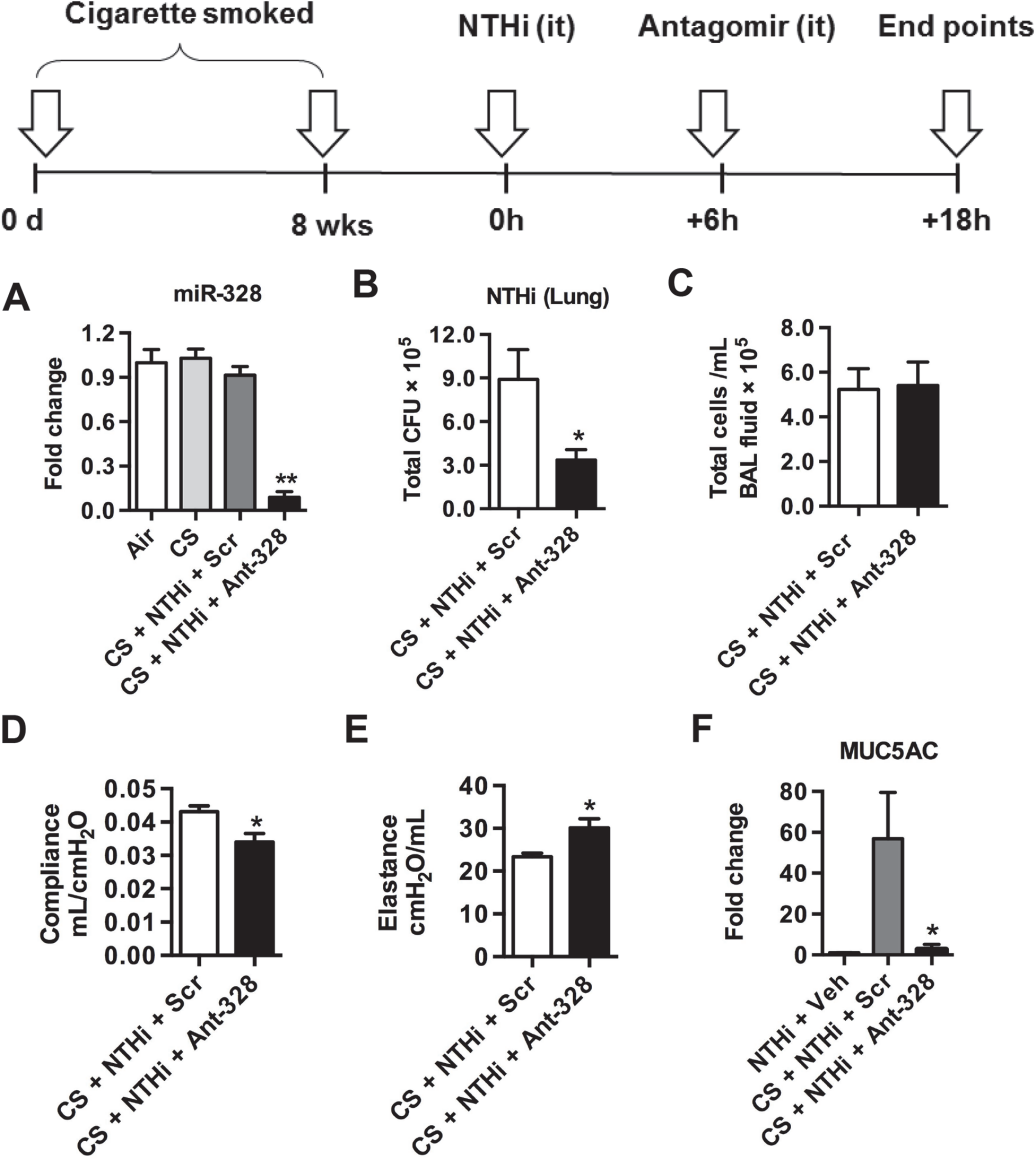
Inhibiting miR-328 enhances bacterial clearance in cigarette smoke-exposed mice. BALB/c mice were exposed to cigarette smoke daily for 8 weeks. At the completion of the model, mice were challenged with NTHi and treated with ant-328 or scrambled antagomir control 6 h post infection. (A) MiR-328 expression in lungs was determined using Taqman PCR, normalised to sno-202 and expressed as fold change compared to vehicle control (B) Bacterial load in the lungs was measured 18 h later by plating and colony counting of lung homogenates, (C) BAL fluid was collected, cells isolated by cytospin and stained, and the total cellular infiltrate was assessed. (D) Compliance and (E) elastance were measured using the flexivent system. (F) Expression of muc-5ac in lung tissue was assessed using quantitative PCR, normalised to HPRT housekeeping control and expressed as fold change over the NTHi + vehicle control. Results are expressed as mean ± SEM (n = 6 mice per group; * p<0.05, ** p<0.01 compared to smoking + NTHi + Scr).

NTHi is commonly isolated from the airways of COPD patients and is linked to exacerbations of disease [20]. Thus, we next investigated if inhibition of miR-328 improved bacterial clearance in our model of cigarette smoke-induced emphysema. Mice were exposed for cigarette smoke for 8 weeks and were then inoculated with NTHi followed by antagomir treatment (scheme Fig 5). Exposure of the lung to ant-328 resulted in significant reduction in the levels of miR-328 (Fig 5A). Again, treatment with ant-328 during infection enhanced NTHi clearance in cigarette smoke-exposed mice (Fig 5B). Total numbers of cellular infiltrates were similar between ant-328 treated and Scr control groups (Fig 5C). In the same model, pulmonary function was measured using the forced oscillation technique. Inhibition of miR-328 function also resulted in a decrease in pulmonary compliance (Fig 5D) and an increase in pulmonary elastance (Fig 5E). Inhibition also nearly completely ablated the increase in muc-5ac expression induced by smoke-exposure with NTHi infection (Fig 5F).

### MiR-328 regulates phagocytosis by human macrophages and neutrophils

**Fig 6:**
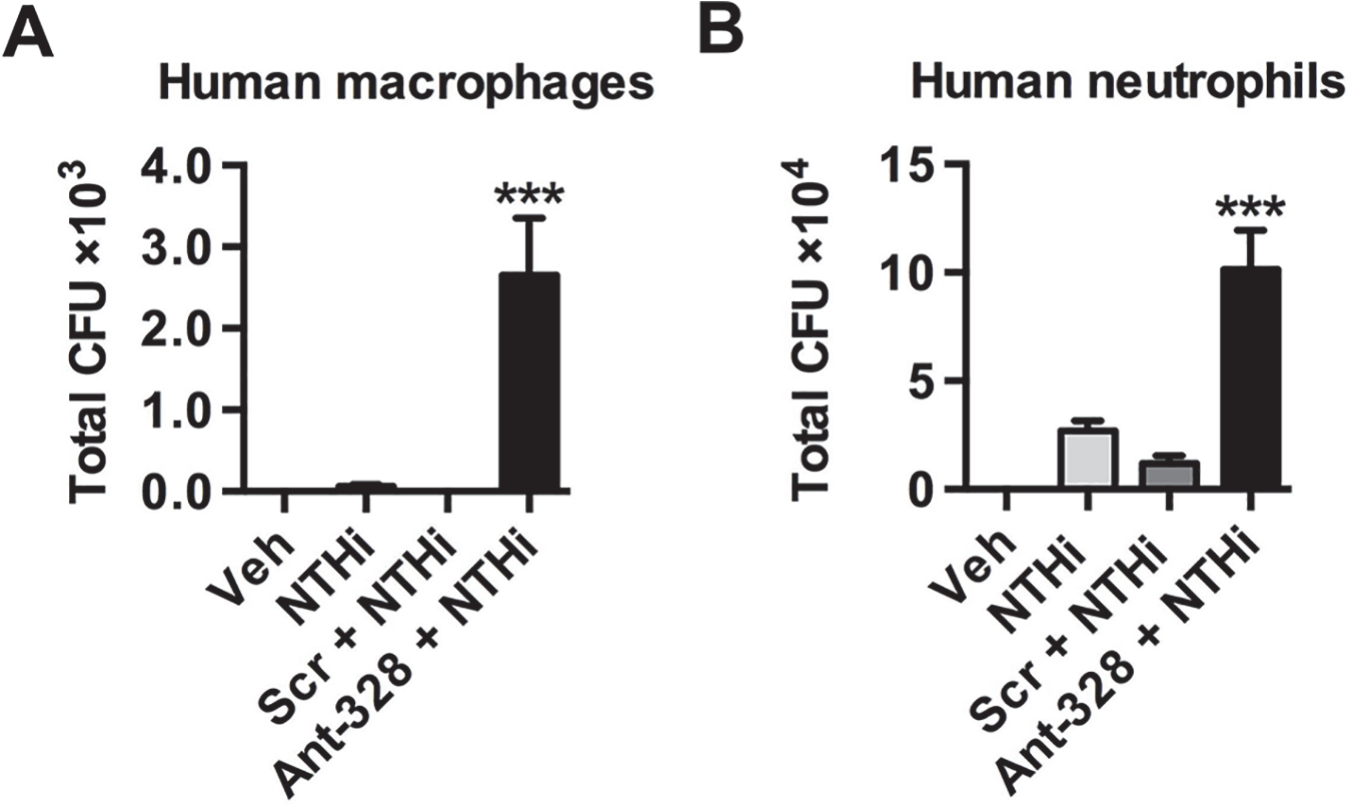
Inhibiting miR-328 increases bacterial uptake in human monocyte-derived macrophages and neutrophils. Human monocytes and neutrophils were isolated from healthy adult blood and macrophages were differentiated *in vitro*. Cells were pre-treated with ant-328 or a scrambled control. (A) Human monocyte-derived macrophages were infected with NTHi for 8 h at MOI 100. (B) Human neutrophils were infected with NTHi for 1 h at MOI 10. Intracellular bacteria colony counts were determined using the gentamicin exclusion assay. Results are expressed as mean ± SEM (n = 6 human subjects per group; *** p<0.001 compared to Scr + NTHi).

MiR-328 is conserved across species, with an identical sequence in mice and humans. Therefore, we investigated if miR-328 plays a similar role in human macrophages and neutrophils infected with NTHi *in vitro*. Neutrophils were purified from healthy adult blood and macrophages were differentiated in culture from monocytes derived from the PBMC fraction. Similar to the results observed with murine cells, inhibition of miR-328 significantly increased bacterial uptake by human macrophages (Fig 6A) and neutrophils (Fig 6B) *in vitro*.

## Discussion

miRNAs are known to be involved in regulating innate immune responses. Expression is induced by various stimuli such as TLR pathways [27,28] or pro-inflammatory cytokines [29,30] and they can act as both positive and negative feedback signals to control these pathways. Furthermore, host pathogen interactions and activation of the immune system has been shown to alter, and be altered by, miRNA expression in various infection models [31–35]. In this study we show that within 24 h of NTHi infection the levels of miRNA in the lung are rapidly altered with expression of individual miRNAs being both increased and decreased. Alterations in the level of expression are strongly associated with bacterial clearance and recruitment of innate immune cells into the lung. Notably from profiling data, miR-328, a highly conserved miRNA, was significantly down-regulated by infection and its basal level are amongst the highest of all down-regulated miRNA, suggesting a key role in the host response to NTHi infection. Similarly, infection of primary lung macrophages by NTHi also downregulated miR-328 expression *in vitro*.

miR-328 is involved in cancer [36–38], autoimmunity [39], and neuronal disease [40]. Here we demonstrate a new role in regulating innate immune cell function, wherein suppression of miR-328 improves bacterial clearance in the lungs. Inhibition of this miRNA in either macrophages or neutrophils from both mice and humans *in vitro* increases uptake (of live and heat-killed NTHi) and decreases survival of bacteria. This indicates that miR-328 regulates phagocytosis and killing of bacteria. Bacterial clearance is also dependent on ROS production [41]. In chronic granulomatous disease, phagocytic activity is normal, however, there is an inability of phagocytes to form ROS after phagocytosis, which leads to increased bacterial survival [42]. Here we show that increased production of ROS in ant-328 treated phagocytes, indicating that this bacterial killing mechanism is also regulated by miR-328. Oxygen-independent bacterial killing mechanisms (such as Cathepsin D) also operate following phagocytosis although they usually coincide with oxygen-dependent lysosomal killing. Cathepsin D is a lysosomal cationic protease that disrupts bacterial membranes, and increases susceptibility of gram-negative bacteria to lysis by lysozyme [43]. We observed increased cathepsin D expression in ant-328 treated macrophages suggesting that bacterial killing was also enhanced using an oxygen independent pathway. Thus miR-328 plays a critical role in the microbial host defence mechanism of innate immune cells by augmenting phagocytosis, the production of ROS and microbicidal activity. Although inhibition with an antagomir dramatically promoted NTHi clearance, application of a mimetic (increasing miR-328 levels) failed to delay clearance of NTHi. We speculate that this is because the levels of endogenous miR-328 are very high, hence masking the effect of mimetic treatment. High levels of miR-328 are linked to macrophage homeostasis and thus it is only when levels are dramatically decreased by antagomir treatment that the cell becomes activated to clear bacteria.

In cancer, miR-328 expression is regulated through the ERK1/2 pathway [38]. Here we found that p38 and JNK MAPK is activated by NTHi infection, and inhibition of these MAPK signalling prior to bacterial infection prevents the down-regulation of miR-328 levels. This suggests that NTHi suppresses miR-328 expression via activation of the p38 and JNK MAPK pathway. We did not observe a functional role for ERK. These experiments were performed *in vitro* with macrophages. Thus, it is possible that miR-328 expression could be regulated by other pathways in a more complex *in vivo* environment.

Adoptive transfer of miR-328-depleted macrophages or neutrophils increased bacterial clearance in the lung, further supporting our *in vitro* observations and directly demonstrating that inhibition of miR-328 in these cells amplifies their ability to clear respiratory infections. We next explored the potential of ant-328 as a therapeutic treatment for bacterial lung infection by directly administering the inhibitor to the lung after NTHi inoculation. Inhibition of miR-328 with ant-328 substantially increased bacterial clearance suggesting that targeting this miRNA could be potentially used as a new approach to anti-microbial therapy. Although we detected increased ROS production *in vitro* following ant-328 treatment, which is often linked to lung injury and activation of pro-inflammatory pathways, we did not observe any increase in inflammation as measured by cellular infiltration and the production of pro-inflammatory cytokines in the lung. One potential explanation is that the increased ROS production that occurs with more rapid bacterial clearance following ant-328 treatment does not reach a threshold level or period of production that is required to elicit deleterious inflammatory changes in the lung. Thus, importantly, treatment with ant-328 *in vivo* enhanced bacterial clearance without a consequent negative impact on lung inflammation (cellular recruitment or proinflammatory mediator production) or pathology.

In chronic lung disease, corticosteroid treatment can lead to immunosuppression and increase the risk of pneumonia [23]. In our current study pre-treatment with dexamethasone prior to NTHi infection significantly impaired bacterial clearance from the lungs. Previous studies have shown that dexamethasone inhibits macrophage phagocytosis *in vitro* while treatment *in vivo* suppresses clearance of bacteria [24,25]. Importantly, dexamethasone administration did not alter miR-328 expression levels despite the increased bacterial load in the lungs. Notably, ant-328 treatment of dexamethasone immune-suppressed mice brought about a dramatic reduction in bacterial load, to levels below that seen in immune competent mice. This data demonstrates that although dexamethasone treatment inhibits phagocytosis along with other inflammatory anti-bacterial pathways, this effect is overcome by treatment with ant-328. These results suggest that it may be possible to use ant-328 in conjunction with dexamethasone in the treatment of chronic lung diseases in order to better control bacterial infection. This would be of particular interest in the case of antibiotic resistant bacterial strains where conventional therapies tend to fail and in patients receiving steroid therapy where infections are difficult to control.

Bacterial colonisation and infection are commonly associated with COPD and exacerbation of disease, and NTHi is one of the most frequently isolated strains of bacteria [20]. The reason underlying this may be that alveolar macrophages from COPD patients exposed to cigarette smoke extract are less efficient at phagocytosing NTHi [44,45]. Using a cigarette smoked-induced model of experimental COPD [46], we demonstrated that inhibiting miR-328 brought about a 3-fold increase in bacterial clearance. A definitive characteristic of COPD is the loss of elastic recoil in the lung and increased lower airway remodelling including mucous cell hyperplasia [47]. Following infection with NTHi, treatment with ant-328 significantly improved both elastic recoil and suppressed muc-5ac expression in the lungs of chronically cigarette exposed mice. The exact mechanism of how this occurs remains to be elucidated but it may involve altered surface tension in lungs by impairing mucus production [48]. In a clinical study on acute exacerbation of COPD, half of the sputum samples from patients enrolled tested positive for bacterial growth. Approximately 50% of the strains isolated were *H*. *influenzae* and *Moraxella catarrhalis*, of which the vast majority were resistant to penicillin [49]. MiR-328 has a highly conserved sequence between mice and humans. Inhibition of miR-328 function in human macrophages and neutrophils using ant-328 also resulted in increased bacterial phagocytosis, indicating that our studies are potentially translatable into anti-microbial innate host defence pathways in human cells.

Our study identifies a potential alternative approach to the treatment of pathogenic microbial infections and bacterial induced exacerbation of chronic lung disease. Although the specific targets of miR-328 remain to be elucidated it is likely that this miRNA targets transcripts involved in regulating phagocytosis and controlling the microbicidal activity of the macrophages and neutrophils. Importantly, ant-328 enhances bacterial killing by specifically augmenting bacterial clearance pathways without further promoting a proinflammatory environment that may be deleterious to lung tissue. Targeting miRNA would be of particular interest to enhance therapeutic outcomes in patients suffering from disorders such as COPD, cystic fibrosis and asthma where innate host defences may be compromised due to steroid therapy or underlying disease mechanisms. Innate immune defects also occur in HIV and transplantation patients. In HIV patients, phagocytosis and respiratory burst are reduced in monocytes and neutrophils [50]. After solid organ transplantation ~50% of deaths are due to infection and during the first four months following bone marrow transplantation, phagocytosis by alveolar macrophage phagocytosis is impaired [51,52]. Although speculative, targeting miR-328 may be more broadly applicable and lead to improvement of innate immune cell function in these immune-deficient patients. While the employment of direct inhibitors of miRNA pathways needs to be approached with caution, we would envisage acute application of antagomir to treat infection or exacerbations induced by acute infections. It will be important, however, to study the chronic impact of altering miR-328 function in murine models, and subsequently in human disease. To date we have not observed toxic effects of antagomirs in chronic models of asthma [53]. In a similar approach, treatment of chronically infected chimpanzees with a locked nucleic acid–modified oligonucleotide (SPC3649) complementary to miR-122 induced long-lasting suppression of HCV viremia, with no evidence of viral resistance or side effects in the treated animals [54]. However, continued research into the amazing regulatory powers of these small non-coding RNA is required to fully determine their potential for the treatment of disease and their fundamental role in maintaining health.

This study is the first to identify a role for miR-328 in regulating bacterial infection in the lung and provides proof-of-principle data that targeting specific host miRNAs could lead to future therapeutics to combat multi-drug resistant bacteria and infection in chronic lung diseases and immune-compromised patients.

## Materials and Methods

### Ethics statement

The animal protocols used were conducted in accordance with the NSW, Australia Animal Research legislation. The experimental protocols 987, A-2009-152, A-2009-141 have been reviewed and approved by University of Newcastle Animal Ethics Committee. All adult subjects provided their informed written consent to participate in the study. All human studies were conducted in accordance with approval from the University of Newcastles Human Research Ethics Committee.

### Mice and NTHi

Specific pathogen-free adult male BALB/c mice were used throughout, and were obtained from the University of Newcastle central animal house. NTHi biotype II (NTHi-289) was obtained, prepared and used as previously described [55,56]. Bacteria were prepared prior to infection by growing overnight on chocolate agar plates (Oxoid, Australia) at 37°C in an atmosphere of 5% CO_2_. The following day, the bacteria were harvested, washed and diluted in sterile PBS and CFU calculated by spectrophotometric analysis. Live NTHi were used in NTHi infection both *in vivo* and *in vitro*. Heat killed bacteria were generated by incubating the live NTHi at 70°C for 30min.

### NTHi infection *in vivo*

Mice were anaesthetised (12.5 mg/kg Alfaxan, Jurox, NSW, Australia) intravenously *via* the tail vein and innoculated intratracheally (i.t) using a cathether (Terumo, Sureflo Hospital Supplies of Australia) with 5x10^5^ or 5x10^6^ CFU of live NTHi in 30 μl of PBS. Following NTHi inoculation, mice were sacrificed at various time points and BAL fluid and lung homogenate (homogenised in 1 ml sterile PBS (Gibco, Invitrogen, New Zealand)) were used to determine bacterial growth. Serial dilutions were loaded onto chocolate agar plates (Oxoid, Australia) and incubated overnight at 37°C in an atmosphere of 5% CO_2_. After 16 h, bacterial colonies for NTHi were counted. BAL cells were enumerated as described previously [57].

### miRNA quantitative RT-PCR

Total RNA was isolated using TRI Reagent (Ambion). miRNA expression was validated using TaqMan miRNA assays (Applied Biosystem) according to the manufacturer’s protocol and normalised to the housekeeping small RNA gene sno-202.

### Measurement of respiratory burst by flow cytometry

Macrophages in cell culture were detached in citric saline (0.135 M KCL, 0.015 M Na citrate) while neutrophils were removed by rinsing. Cells were spun down and resuspended in FACS buffer (2% fetal calf serum (FCS; Sigma-Aldrich) in PBS) before incubation with 10μM dihydroethidium (DHE; Sigma-Aldrich) for 30 minutes at 37°C. Fluorescence intensity was measured by flow cytometry with 488nm excitation and 610nm emission.

### Immunofluorescence staining

Immunofluorescence assays were performed as described previously [56]. Macrophages were labelled with 5μM of CFSE (Molecular Probes) for 10 minutes at 37°C. Staining was quenched with fetal calf serum (FCS) for 1 minute, and cells were washed and resuspended in PBS. Cells were fixed in 1% (w/v) paraformaldehyde in PBS buffer for 10 min at room temperature (RT), blocked and permeabilized with 0.2% (v/v) Triton X-100 and 5% normal goat serum for 1 h at RT. Cathepsin D (CTSD) expression were stained using rabbit monoclonal Ab to CTSD (Abcam) overnight at 4°C. Cells were then incubated with Cy3-conjugated goat anti-rabbit IgG (GE Healthcare) for 45 minutes at RT and nucleus was counter-stained with DAPI (Sigma-Aldrich). Images were analyzed using Olympus FluoView FV1000 confocal microscope.

### Inhibition of p38 MAPK signalling pathways

Macrophages were pre-treated 5μM doramapimod (inhibit p38 MAPK) (LC lab), 50μM SP600125 (inhibit JNK) (LC lab), 50μM U0126 (inhibit ERK) (LC lab) for 30 minutes,prior to NTHi infection for 8 h. DMSO was used as a vehicle control.

### Cigarette smoke exposure

Mice were exposed to cigarette smoke (in the form of 12 cigarettes) in a closed chamber twice a day, 5 days per week for a duration of 8 weeks as previously described [46].

### Dexamethasone treatment

Mice were injected intraperitoneally (i.p.) with 3mg/kg of dexamethasone (DEX; Sigma-Aldrich) for 3 consecutive days before they were inoculated with (5 x 10^5^ CFU / mouse) with NTHi.

### Antagomir oligonucleotides

The complementary antagomir strand of the miR-328-3p sequence obtained from miRbase was synthesised from Sigma-Aldrich with the following chemical modifications 5’mA.*.mC.*.mG.mG.mA.mA.mG.mG.mG.mC.mA.mG.mA.mG.mA.mG.mG.mG.mC.*.mC.*.mA.*.mG.*. 3’-Chol where “m” represents 2’-OMe-modified phosphoramidites, “*” represents phosphorothioate linkages, and “-Chol” was hydroxyprolinol-linked cholesterol to allow permeation of cell membranes. The scrambled control antagomir sequence was 5'mU.*.mC.*.mA.mC.mA.mA.mC.mC.mU.mC.mC.mU.mA.mG.mA.mA.mA.mG.mA.*.mG.*.mU.*.mA.*.3'-Chol. Mice were treated *in vivo* with 50μg of antagomir intratracheally (see NTHi infection: methods), and macrophages and neutrophils were treated with antagomir at 50μg/ml 12 h before assays *in vitro*.

### Statistical analysis

Results are presented as means ± standard errors of the means (SEM). Results were analysed using one way ANOVA plus Bonferroni post-test for statistical significance or Student's t-test plus Mann-Whitney test. Analysis was performed with Prism v4.0 (GraphPad Software, CA. USA). p< 0.05 was considered to represent a significant difference.

Additional methods were described in S1 Methods.

## Supporting Information

S1 Fig Heatmap of miRNAs expression in the airways following NTHi infection. Total RNA from the airways of NTHi infected mice was extracted and miRNA microarrays performed. Differential expressions of miRNA were compared to sham-inoculated control groups (PBS). In the heat map, lowest (blue) and highest (red) expression of miRNAs.
(TIF)

S2 Fig MiR-328 expression following NTHi infection *in vivo*. Mice were inoculated i.t. with 5x10^5^ CFU of NTHi. MiR-328 expression levels in (A) airways and (B) lung macrophages measured using Taqman qPCR normalised to sno-202 and expressed as fold change compared to control. Results are expressed as mean ± SEM. (n = 5–7 mice per group; * p<0.05, ** p<0.01, *** p<0.001, **** p<0.0001 vs. vehicle control)
(TIF)

S3 Fig Inhibition of miR-328 increases phagocytosis and bacterial clearance by neutrophils *in vitro*. Studies were performed using bone marrow neutrophils isolated from BALB/c mice. Neutrophils were pre-treated with ant-328 for 12 h to knockdown expression before infection with NTHi for 1 h. Scrambled antagomir was used as control. (a) MiR-328 knockdown by ant-328 was assessed using Taqman qPCR, normalised to sno-202 and expressed as fold change compared to scrambled antagomir control. (b) Bacteria in the supernatant were measured 1 h post-inoculation by plating and colony counting. (c) Gentamicin exclusion assay was performed by killing extracellular bacteria before cells were lysed to release intracellular bacteria, which were plated and counted. (d) Flow cytometry histograms showing uptake of heat-killed NTHi. NTHi were heat-killed and labelled with CFSE before exposure to neutrophils for 1 h at a MOI of 10. Intensity of CFSE was analysed using flow cytometry. (e) Dihydroethidium and flow cytometry was used to monitor superoxide production in cells 1 h following NTHi inoculation. MFI depicts one experiment representative of 4 independent experiments. (n = 3–4 samples per group; * p<0.05, ** p<0.01, *** p<0.001 compared to Scr + NTHi).
(TIF)

S4 Fig Inhibition of NTHi phagocytosis *in vitro*. Primary lung macrophages were isolated from the lungs of naïve mice and pre-treated with ant-328 or Scr for 12 h before treatment with cytochalasin D or vehicle for 1 h. Bacteria were heat-killed and labeled with CFSE then exposed to macrophages for 1 h. Macrophage intracellular CFSE fluorescence was assessed by flow cytometry. Graph depicts one experiment representative of 3 independent experiments.
(TIF)

S5 Fig No changes in cell viability following ant-328 treatment. (A) Mouse macrophage and (B) neutrophil viability was measured by flow cytometry by gating the percentage of cells that were both Annexin V^-^ and 7’-AAD^-^. Results are expressed as mean ± SEM. (n = 3–4 samples per group)
(TIF)

S6 Fig Effect of miR-328 mimic on bacterial clearance. Primary lung macrophages were isolated from the lungs of naïve mice and pre-treated with miR-328 mimic for 24 h before infection with NTHi at MOI 100. miR-Scr was used as a control. (A) Bacterial numbers in culture supernatants were measured at 8 h post-inoculation by colony counts. (B) Intracellular bacterial counts obtained using gentamicin exclusion assay, whereby gentamicin treatment kills extracellular bacteria prior to cell lysis and remaining intracellular bacteria were plated and counted. Results are expressed as mean ± SEM. (n = 4 samples per group).
(TIF)

S7 Fig Inhibition of miR-328 increased expression of bacterial binding molecules CD14, CD36, and CD11b. Primary lung macrophages were isolated from the lungs of naïve mice and pre-treated with ant-328 for 12 h. Expression of (A) CD14, (B) CD36, (C) CD11b mRNA was assessed using quantitative PCR, normalised to HPRT housekeeping control and expressed as fold change over Scr control. Results are expressed as mean ± SEM. (n = 6 samples per group; *p<0.05, **p<0.01, *** p<0.001)
(TIF)

S8 Fig Effect of miR-328 inhibition on ROS generation. Dihydroethidium and flow cytometry was used to monitor superoxide production in (A) macrophages and (B) neutrophils following antagomir treatment. Results are expressed as mean ± SEM. MFI depicts one experiment representative of 4 independent experiments (***p<0.001 compared to scrambled antagomir control)
(TIF)

S9 Fig Proinflammatory cytokines production *in vitro*. Primary lung macrophages were pre-treated with ant-328 for 12 h before infection with NTHi MOI 100 for 8 h. Culture supernatant was collected and protein levels of (A) IL-6 and (B) TNF-α were determined by ELISA. Results are expressed as mean ± SEM. (n = 6 samples per group)
(TIF)

S10 Fig NTHi activates p38 and JNK signalling. Primary lung macrophages were isolated from the lungs of naïve mice and treated with NTHi for 1 h. Level of (A) Phosphorylated p38 and (C) ERK were assessed by flow cytometry. (B) Phosphorylated JNK was measured using ELISA. Expression of pJNK was not detected (N.D) in vehicle control group. Results are expressed as mean ± SEM. (n = 3–4 samples per group; ***p<0.001 compared to vehicle)
(TIF)

S11 Fig Inhibition of various miRNAs on bacterial clearance *in vitro*. Neutrophils were treated with various antagomirs (as indicated) to knockdown expression of the corresponding miRNA and extracellular bacteria in the supernatant were measured 1 h post-infection by colony count. Results are expressed as mean ± SEM. (n = 3 samples per group)
(TIF)

S12 Fig Adoptive transfer of antagomir-treated macrophages and neutrophils into the lungs of naïve recipient BALB/c mice. MiR-328 was inhibited in macrophages or neutrophils with ant-328 for 12 h *ex vivo*. Scrambled antagomir was used as a control. (A, D) Naïve mice were not administered any cells. (B-C) Macrophages or (E-F) neutrophils were labelled with CFSE and adoptively transferred i.t. into naïve mice. The presence of adoptively transferred labelled macrophages and neutrophils in the lungs of recipient mice was assessed by flow cytometry.
(TIF)

S13 Fig Cellular infiltration in lungs following ant-328 treatment. MiR-328 in (A-E) macrophages or (F-J) neutrophils was inhibited with antagomir for 12 h *ex vivo* and adoptively transferred i.t. into naïve mice before the mice were infected with NTHi. (K-N) In a different model, naïve mice were inoculated with NTHi for 6 h before treated with ant-328 or scrambled antagomir i.t. (A, F) Total cellular infiltrate in BAL fluid was enumerated by cell counts. Cells in BAL fluid were then cytospinned and stained with May-grunwald. Differential cell counts were performed to identify numbers of (B, G, K) macrophages and (C, H, L) neutrophils based on cell morphology. Lung homogenates were used to determine protein levels of (D, I, M) IL-6 and (E, J, N) TNF-α by ELISA. Results are expressed as mean ± SEM. (n = 4–6 mice per group; * p<0.05 compared to scrambled antagomir control).
(TIF)

S14 Fig Effect of miR-328 on lung inflammation in non-infected mice. Naïve mice were treated with antagomirs for 12 h. (A) Total cellular infiltrate in BAL fluid was enumerated by cell counts. Cells in BAL fluid were then cytospinned and stained with May-Grunwald. Differential cell counts were performed to identify numbers of (B) macrophages and (C) neutrophils. Lung homogenates were used to determine protein levels of (D) TNF-α by ELISA. Results are expressed as mean ± SEM. (n = 5–6 mice per group)
(TIF)

S1 Methods Supplemental methods. (PDF)
