# Correction: Antagonism of miR-328 Increases the Antimicrobial Function of Macrophages and Neutrophils and Rapid Clearance of Non-typeable *Haemophilus Influenzae* (NTHi) from Infected Lung

**DOI:** 10.1371/journal.ppat.1004956

**Published:** 2015-06-24

**Authors:** Hock L. Tay, Gerard E. Kaiko, Maximilian Plank, JingJing Li, Steven Maltby, Ama-Tawiah Essilfie, Andrew Jarnicki, Ming Yang, Joerg Mattes, Philip M. Hansbro, Paul S. Foster

The last two authors, Philip M. Hansbro and Paul S. Foster, should be noted as contributing equally to this work.
