# Supplementary figures and images for: Antagonism of miR-328 Increases the Antimicrobial Function of Macrophages and Neutrophils and Rapid Clearance of Non-typeable Haemophilus Influenzae (NTHi) from Infected Lung
Source: PLoS Pathog. 2015 Apr 20;11(4):e1004549. doi: 10.1371/journal.ppat.1004549 (PMC4404141; doi:10.1371/journal.ppat.1004549)

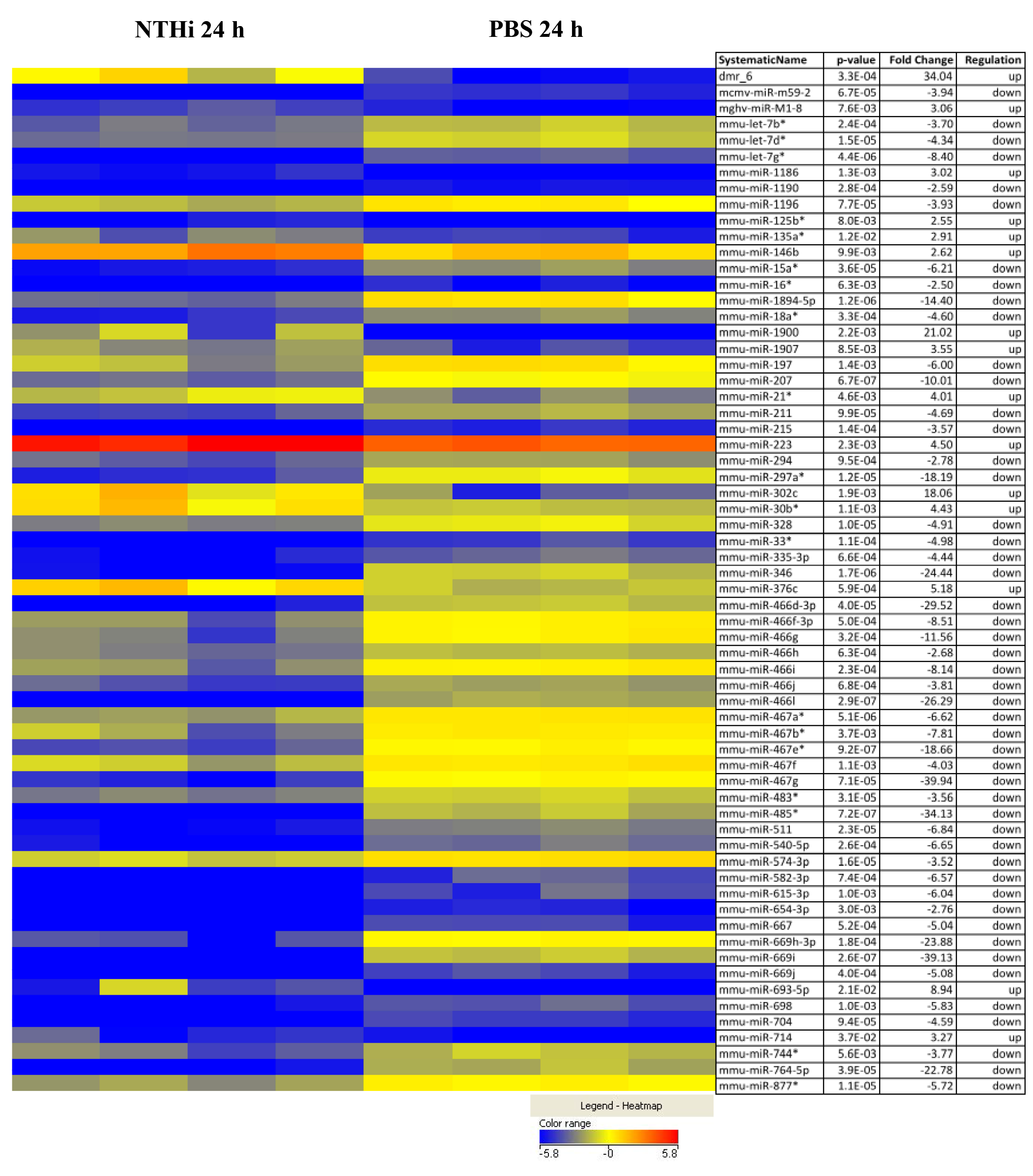

Supplement: S1 Fig — Total RNA from the airways of NTHi infected mice was extracted and miRNA microarrays performed. Differential expressions of miRNA were compared to sham-inoculated control groups (PBS). In the heat map, lowest (blue) and highest (red) expression of miRNAs. (TIF) [file ppat.1004549.s001.tif]

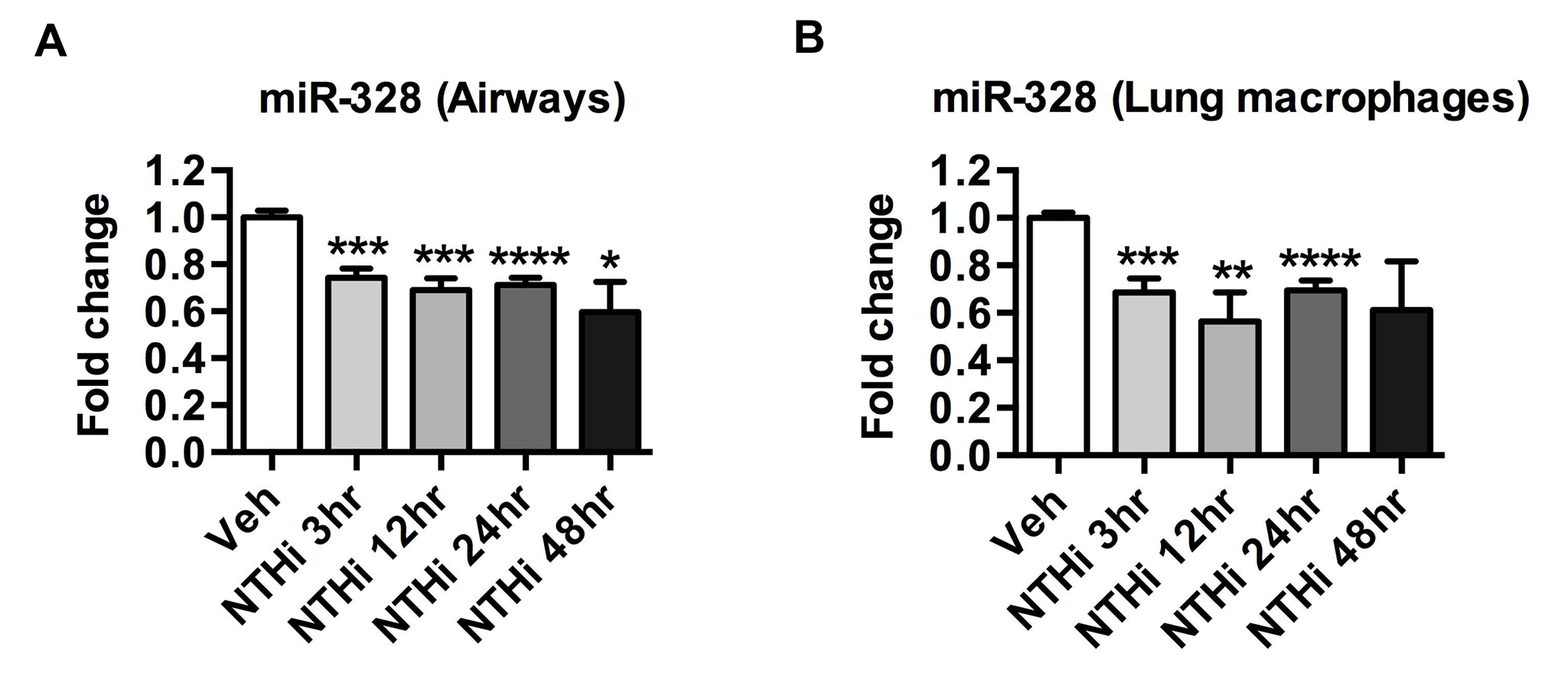

Supplement: S2 Fig — Mice were inoculated i.t. with 5x10^5^ CFU of NTHi. MiR-328 expression levels in (A) airways and (B) lung macrophages measured using Taqman qPCR normalised to sno-202 and expressed as fold change compared to control. Results are expressed as mean ± SEM. (n = 5–7 mice per group; * p<0.05, ** p<0.01, *** p<0.001, **** p<0.0001 vs. vehicle control) (TIF) [file ppat.1004549.s002.tif]

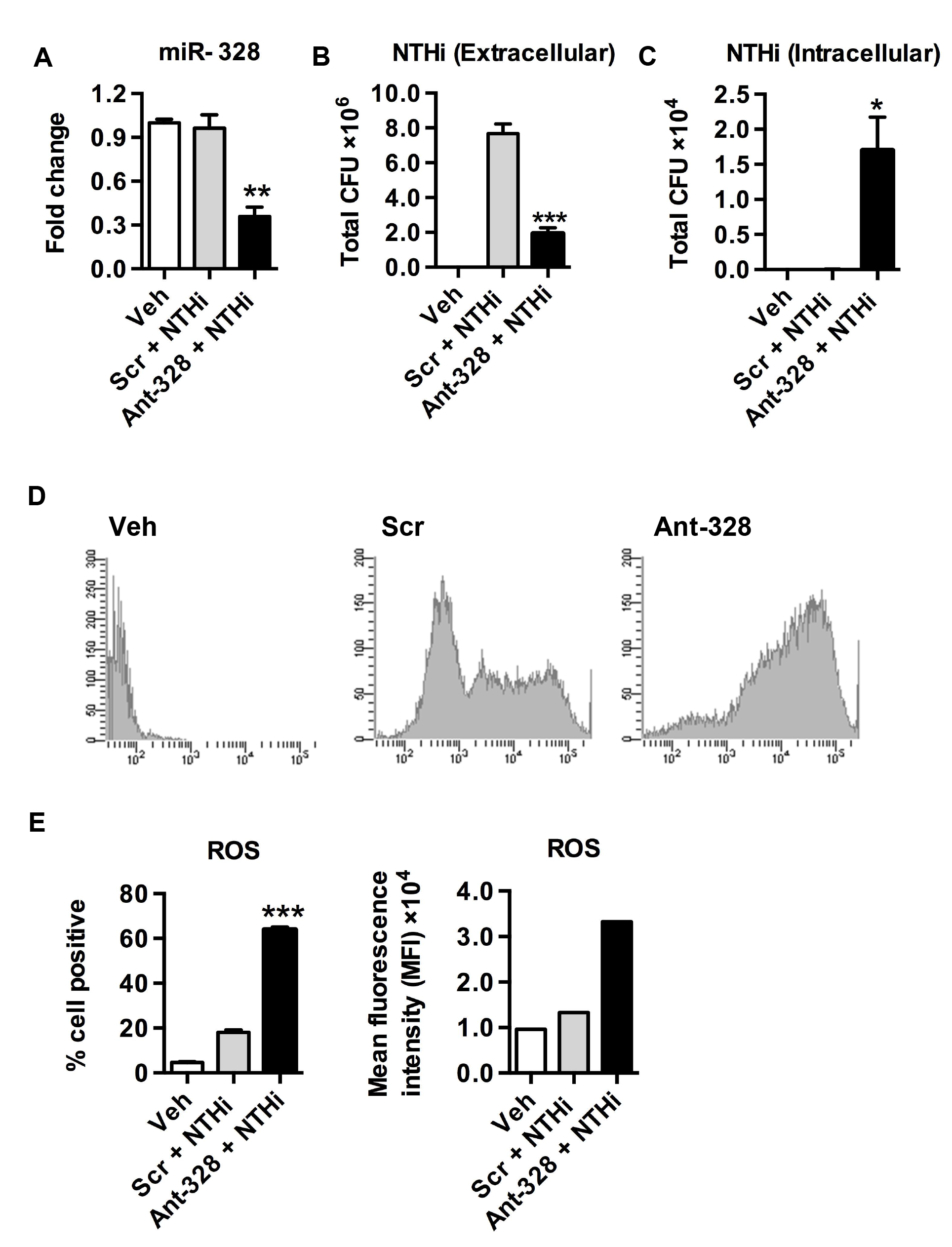

Supplement: S3 Fig — Studies were performed using bone marrow neutrophils isolated from BALB/c mice. Neutrophils were pre-treated with ant-328 for 12 h to knockdown expression before infection with NTHi for 1 h. Scrambled antagomir was used as control. (a) MiR-328 knockdown by ant-328 was assessed using Taqman qPCR, normalised to sno-202 and expressed as fold change compared to scrambled antagomir control. (b) Bacteria in the supernatant were measured 1 h post-inoculation by plating and colony counting. (c) Gentamicin exclusion assay was performed by killing extracellular bacteria before cells were lysed to release intracellular bacteria, which were plated and counted. (d) Flow cytometry histograms showing uptake of heat-killed NTHi. NTHi were heat-killed and labelled with CFSE before exposure to neutrophils for 1 h at a MOI of 10. Intensity of CFSE was analysed using flow cytometry. (e) Dihydroethidium and flow cytometry was used to monitor superoxide production in cells 1 h following NTHi inoculation. MFI depicts one experiment representative of 4 independent experiments. (n = 3–4 samples per group; * p<0.05, ** p<0.01, *** p<0.001 compared to Scr + NTHi). (TIF) [file ppat.1004549.s003.tif]

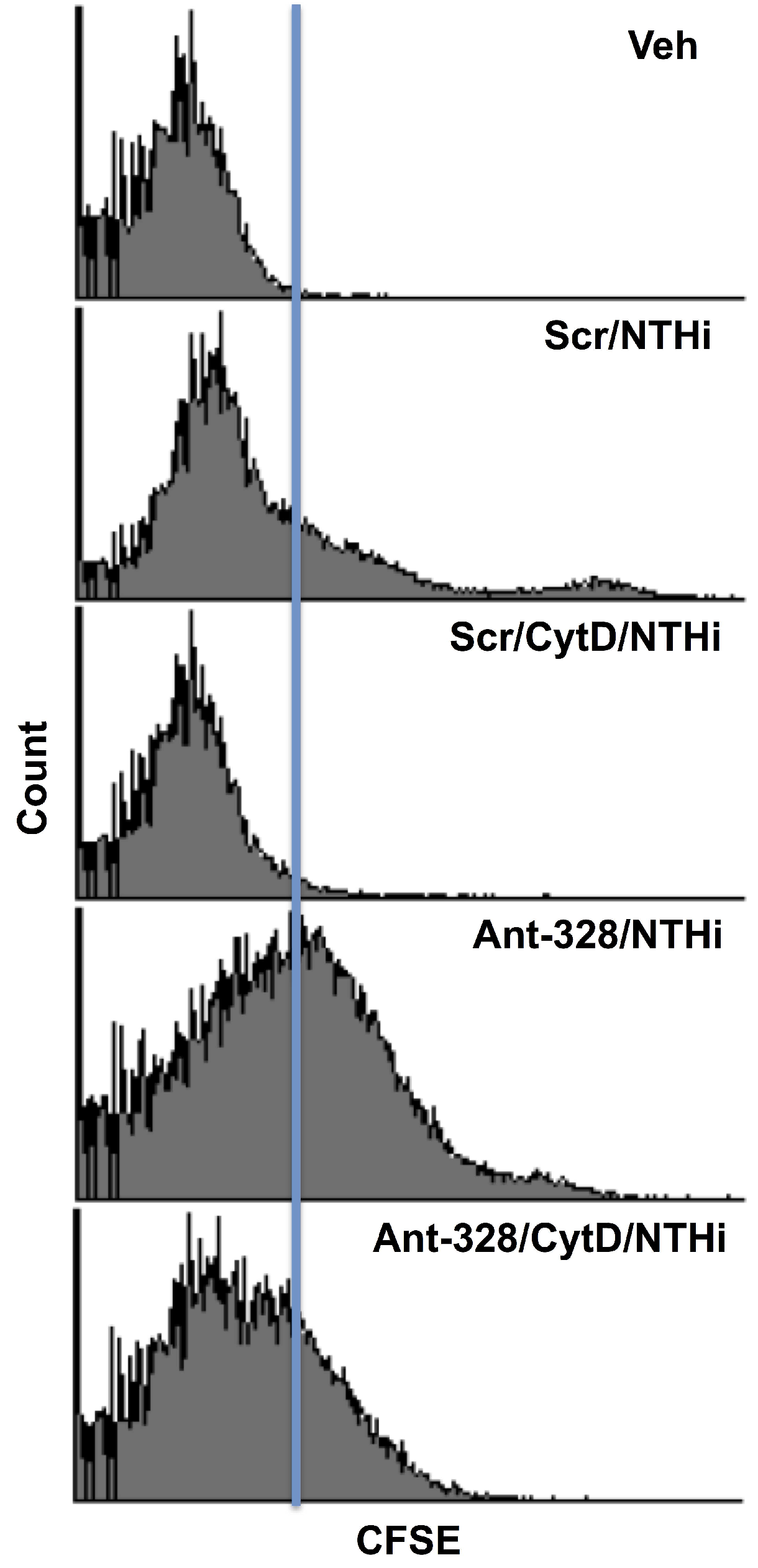

Supplement: S4 Fig — Primary lung macrophages were isolated from the lungs of naïve mice and pre-treated with ant-328 or Scr for 12 h before treatment with cytochalasin D or vehicle for 1 h. Bacteria were heat-killed and labeled with CFSE then exposed to macrophages for 1 h. Macrophage intracellular CFSE fluorescence was assessed by flow cytometry. Graph depicts one experiment representative of 3 independent experiments. (TIF) [file ppat.1004549.s004.tif]

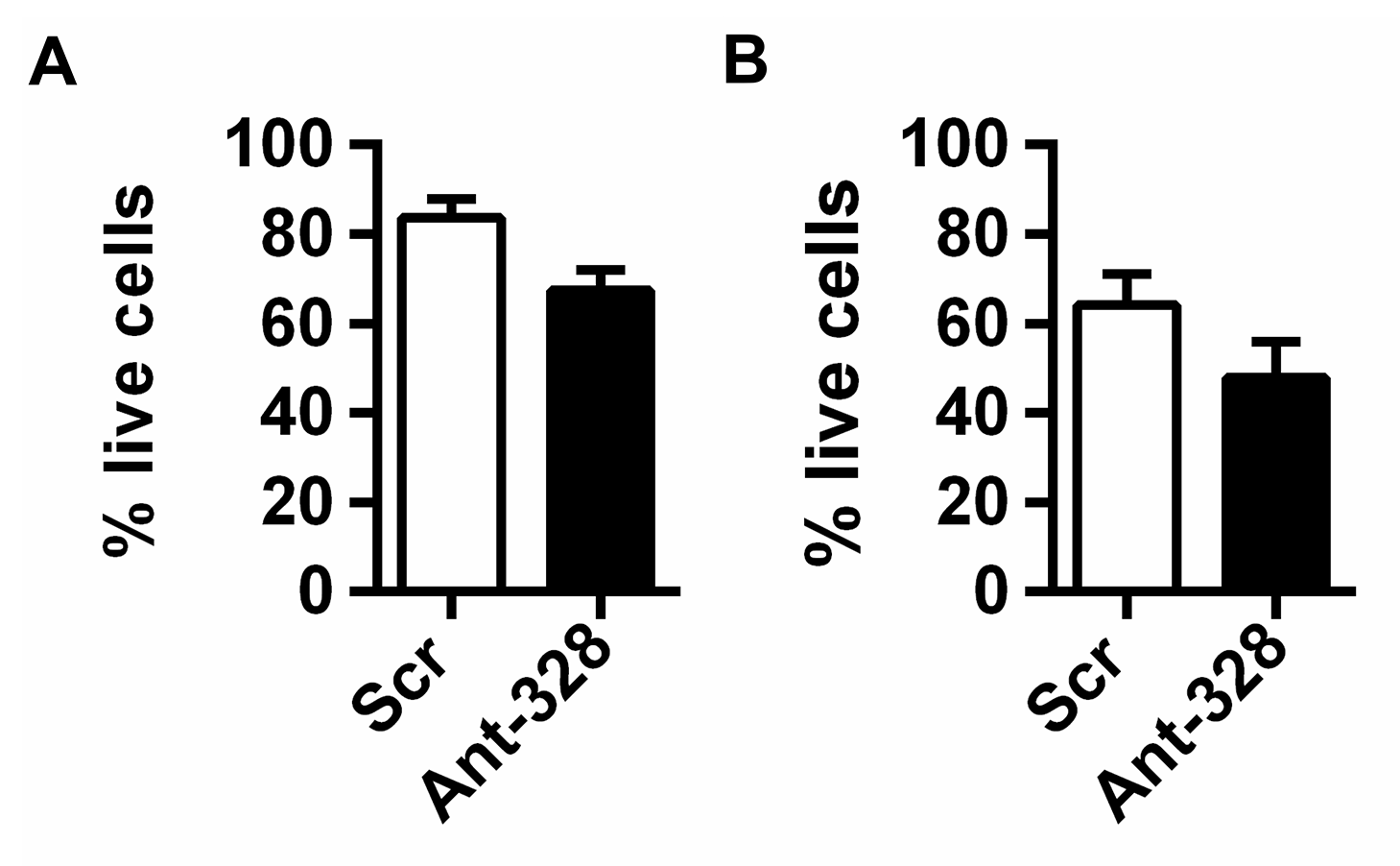

Supplement: S5 Fig — (A) Mouse macrophage and (B) neutrophil viability was measured by flow cytometry by gating the percentage of cells that were both Annexin V- and 7’-AAD-. Results are expressed as mean ± SEM. (n = 3–4 samples per group) (TIF) [file ppat.1004549.s005.tif]

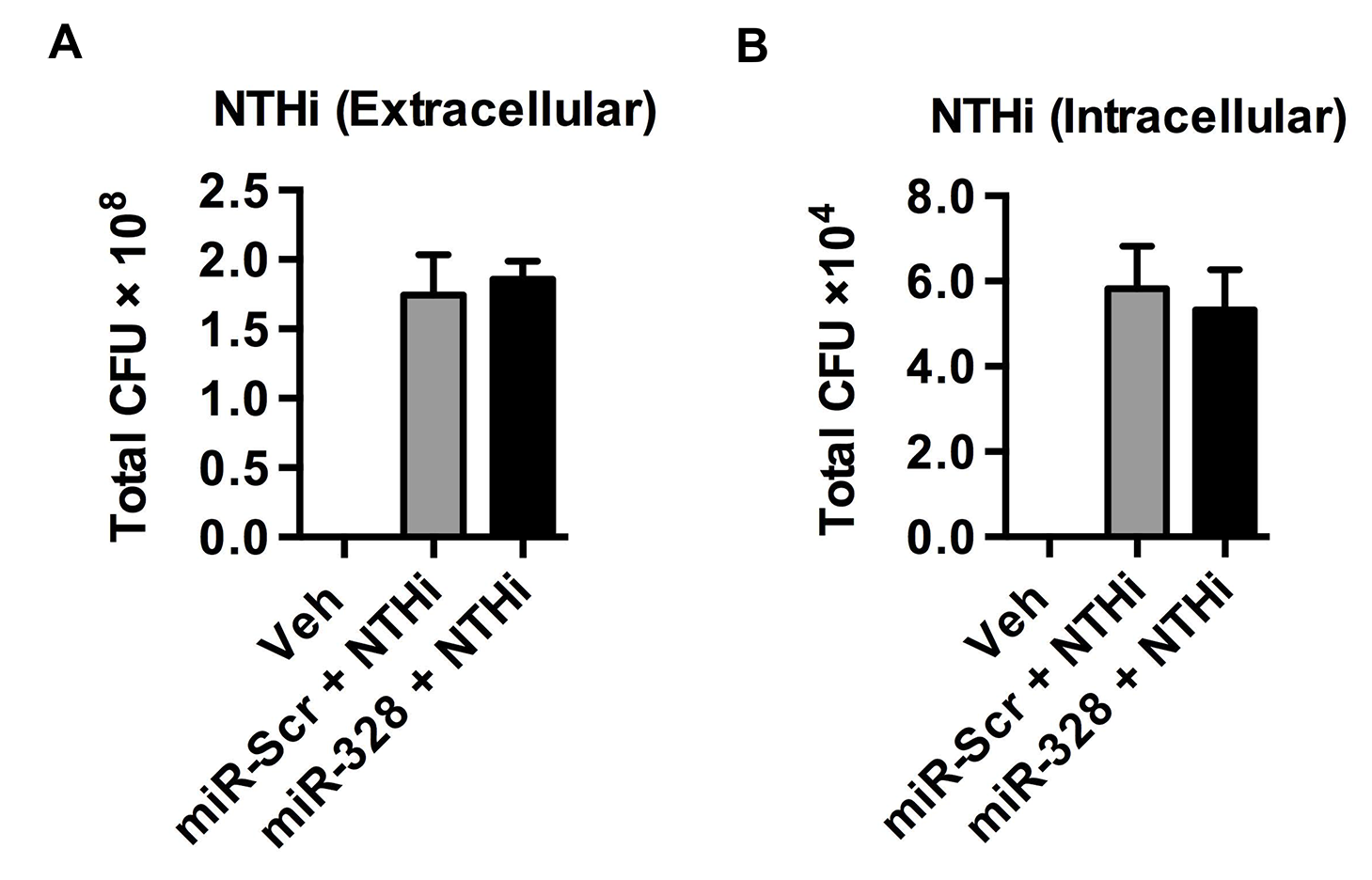

Supplement: S6 Fig — Primary lung macrophages were isolated from the lungs of naïve mice and pre-treated with miR-328 mimic for 24 h before infection with NTHi at MOI 100. miR-Scr was used as a control. (A) Bacterial numbers in culture supernatants were measured at 8 h post-inoculation by colony counts. (B) Intracellular bacterial counts obtained using gentamicin exclusion assay, whereby gentamicin treatment kills extracellular bacteria prior to cell lysis and remaining intracellular bacteria were plated and counted. Results are expressed as mean ± SEM. (n = 4 samples per group). (TIF) [file ppat.1004549.s006.tif]

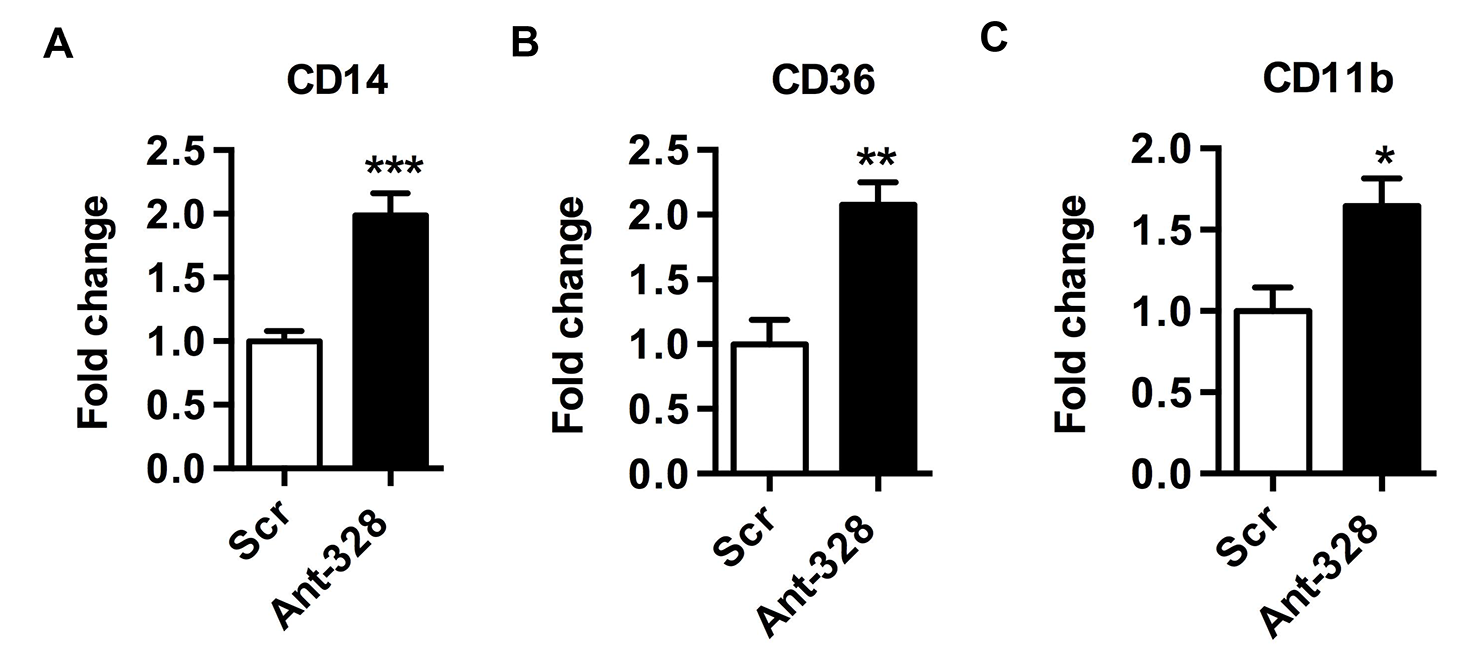

Supplement: S7 Fig — Primary lung macrophages were isolated from the lungs of naïve mice and pre-treated with ant-328 for 12 h. Expression of (A) CD14, (B) CD36, (C) CD11b mRNA was assessed using quantitative PCR, normalised to HPRT housekeeping control and expressed as fold change over Scr control. Results are expressed as mean ± SEM. (n = 6 samples per group; *p<0.05, **p<0.01, *** p<0.001) (TIF) [file ppat.1004549.s007.tif]

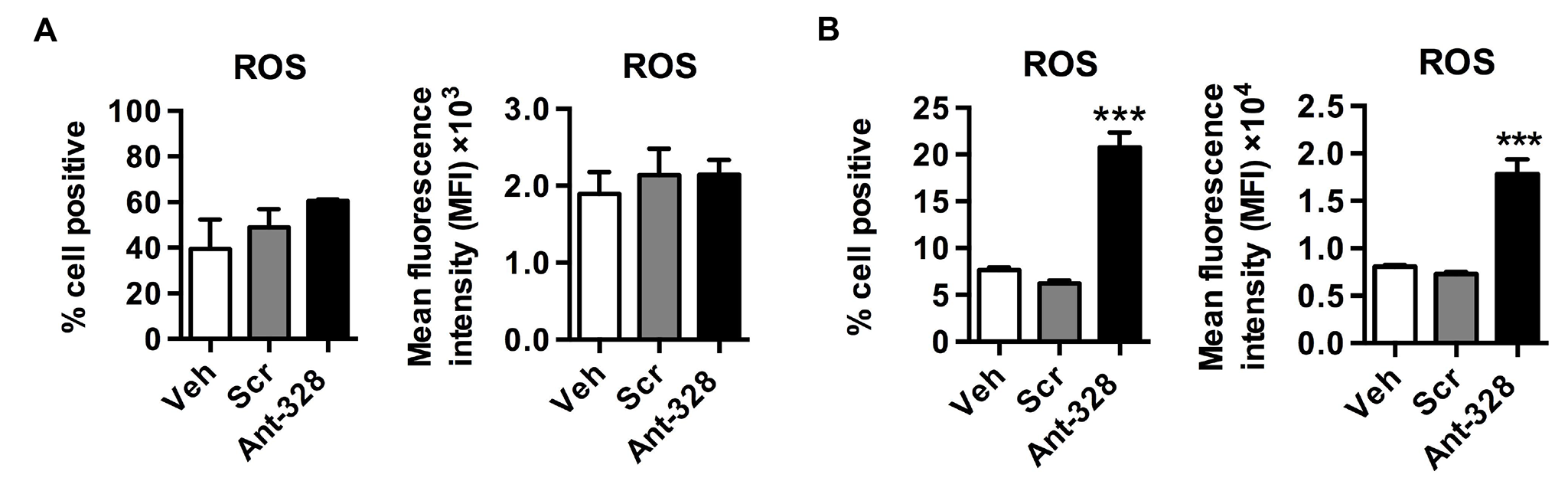

Supplement: S8 Fig — Dihydroethidium and flow cytometry was used to monitor superoxide production in (A) macrophages and (B) neutrophils following antagomir treatment. Results are expressed as mean ± SEM. MFI depicts one experiment representative of 4 independent experiments (***p<0.001 compared to scrambled antagomir control) (TIF) [file ppat.1004549.s008.tif]

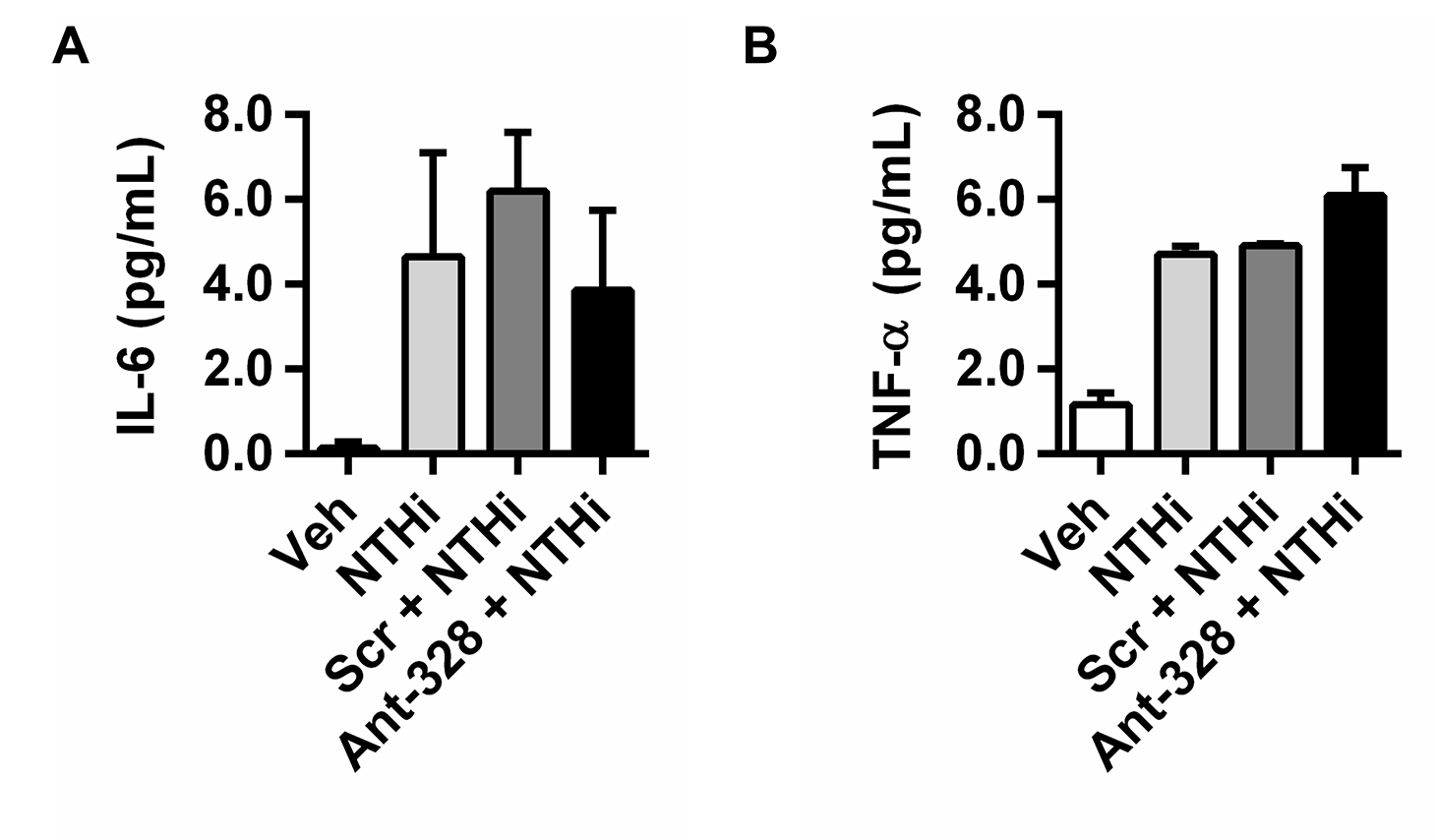

Supplement: S9 Fig — Primary lung macrophages were pre-treated with ant-328 for 12 h before infection with NTHi MOI 100 for 8 h. Culture supernatant was collected and protein levels of (A) IL-6 and (B) TNF-α were determined by ELISA. Results are expressed as mean ± SEM. (n = 6 samples per group) (TIF) [file ppat.1004549.s009.tif]

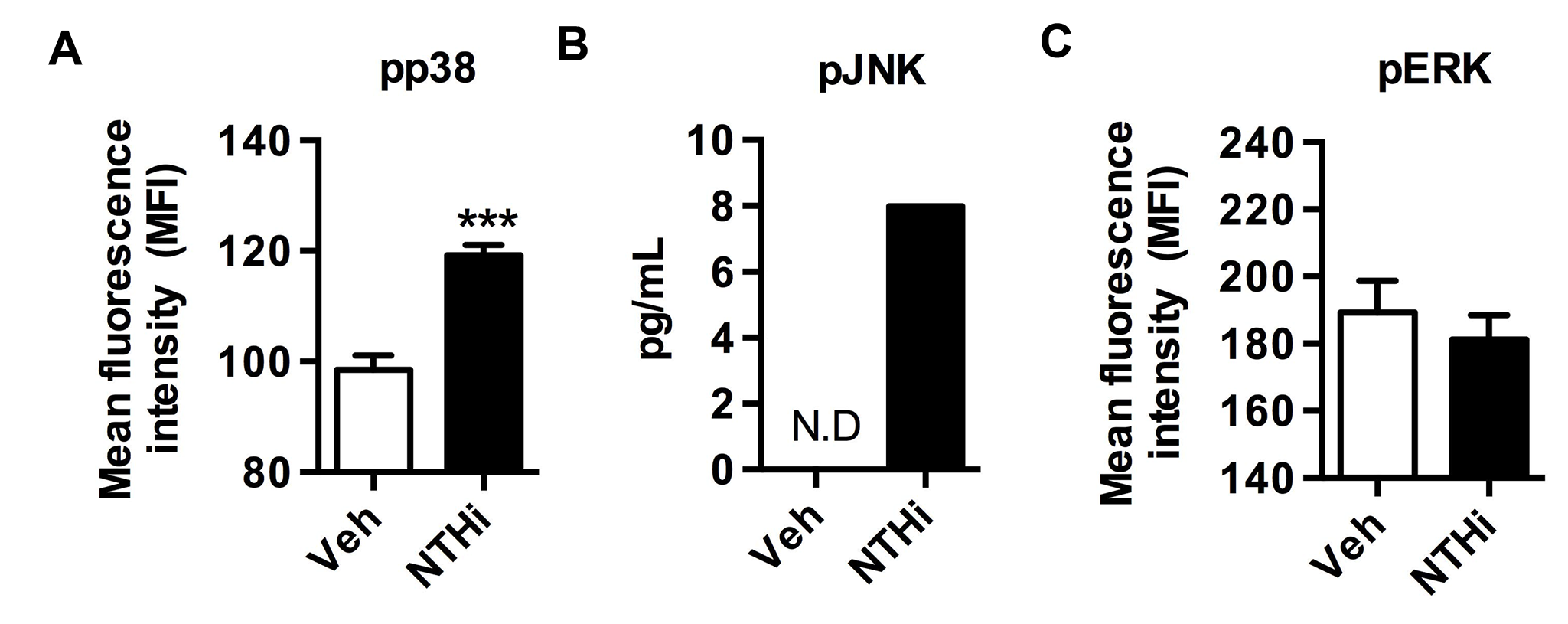

Supplement: S10 Fig — Primary lung macrophages were isolated from the lungs of naïve mice and treated with NTHi for 1 h. Level of (A) Phosphorylated p38 and (C) ERK were assessed by flow cytometry. (B) Phosphorylated JNK was measured using ELISA. Expression of pJNK was not detected (N.D) in vehicle control group. Results are expressed as mean ± SEM. (n = 3–4 samples per group; ***p<0.001 compared to vehicle) (TIF) [file ppat.1004549.s010.tif]

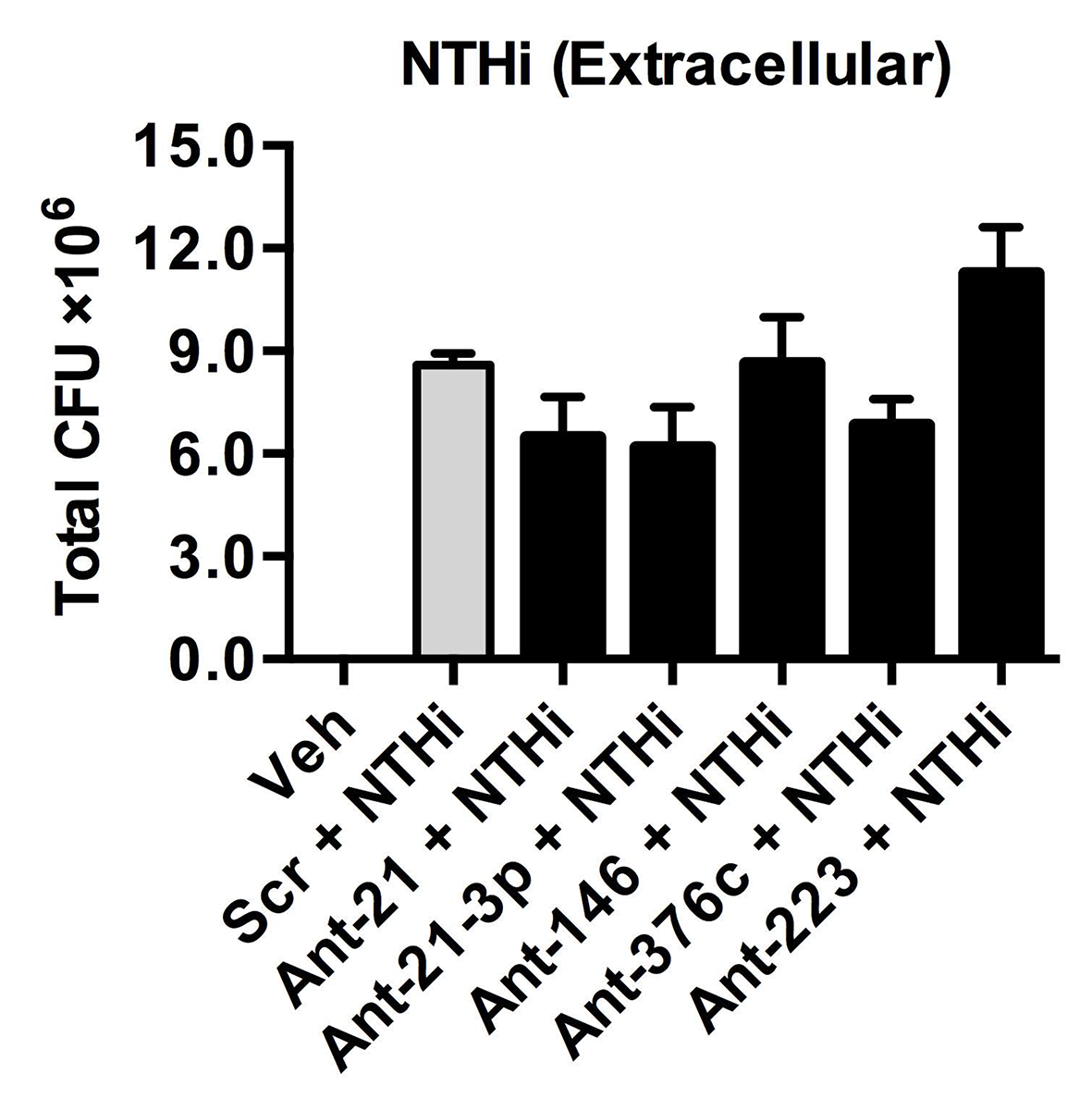

Supplement: S11 Fig — Neutrophils were treated with various antagomirs (as indicated) to knockdown expression of the corresponding miRNA and extracellular bacteria in the supernatant were measured 1 h post-infection by colony count. Results are expressed as mean ± SEM. (n = 3 samples per group) (TIF) [file ppat.1004549.s011.tif]

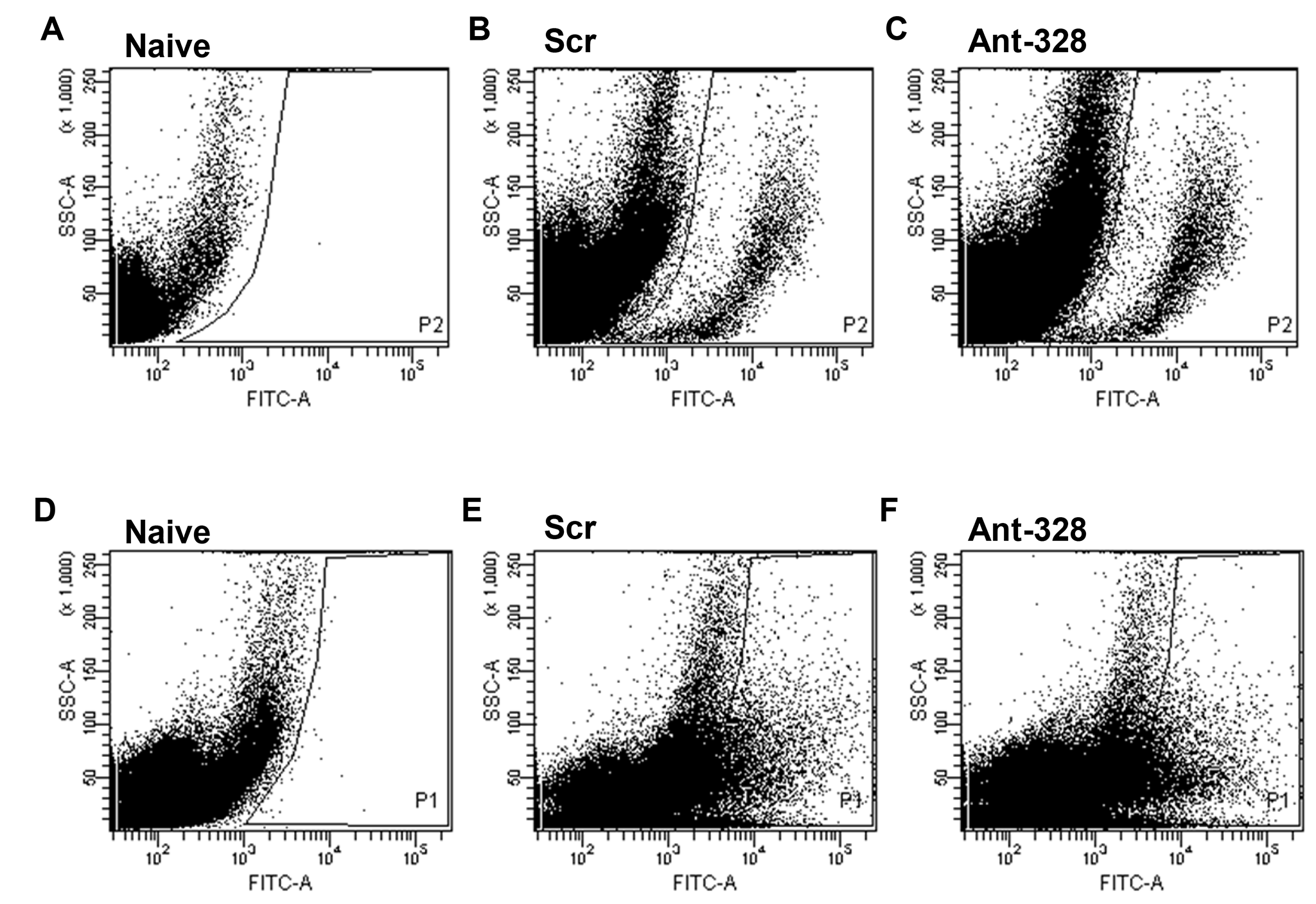

Supplement: S12 Fig — MiR-328 was inhibited in macrophages or neutrophils with ant-328 for 12 h ex vivo. Scrambled antagomir was used as a control. (A, D) Naïve mice were not administered any cells. (B-C) Macrophages or (E-F) neutrophils were labelled with CFSE and adoptively transferred i.t. into naïve mice. The presence of adoptively transferred labelled macrophages and neutrophils in the lungs of recipient mice was assessed by flow cytometry. (TIF) [file ppat.1004549.s012.tif]

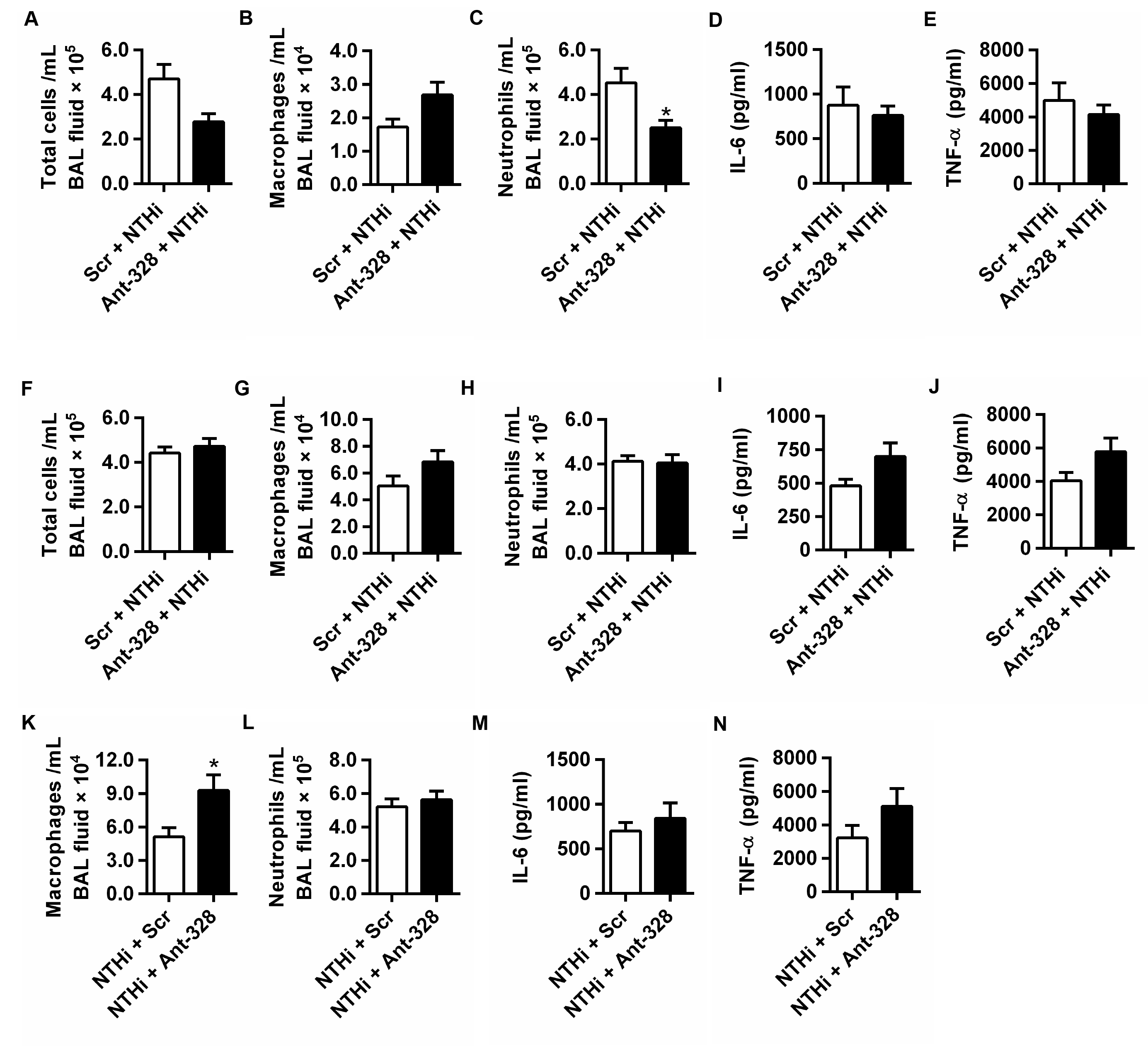

Supplement: S13 Fig — MiR-328 in (A-E) macrophages or (F-J) neutrophils was inhibited with antagomir for 12 h ex vivo and adoptively transferred i.t. into naïve mice before the mice were infected with NTHi. (K-N) In a different model, naïve mice were inoculated with NTHi for 6 h before treated with ant-328 or scrambled antagomir i.t. (A, F) Total cellular infiltrate in BAL fluid was enumerated by cell counts. Cells in BAL fluid were then cytospinned and stained with May-grunwald. Differential cell counts were performed to identify numbers of (B, G, K) macrophages and (C, H, L) neutrophils based on cell morphology. Lung homogenates were used to determine protein levels of (D, I, M) IL-6 and (E, J, N) TNF-α by ELISA. Results are expressed as mean ± SEM. (n = 4–6 mice per group; * p<0.05 compared to scrambled antagomir control). (TIF) [file ppat.1004549.s013.tif]

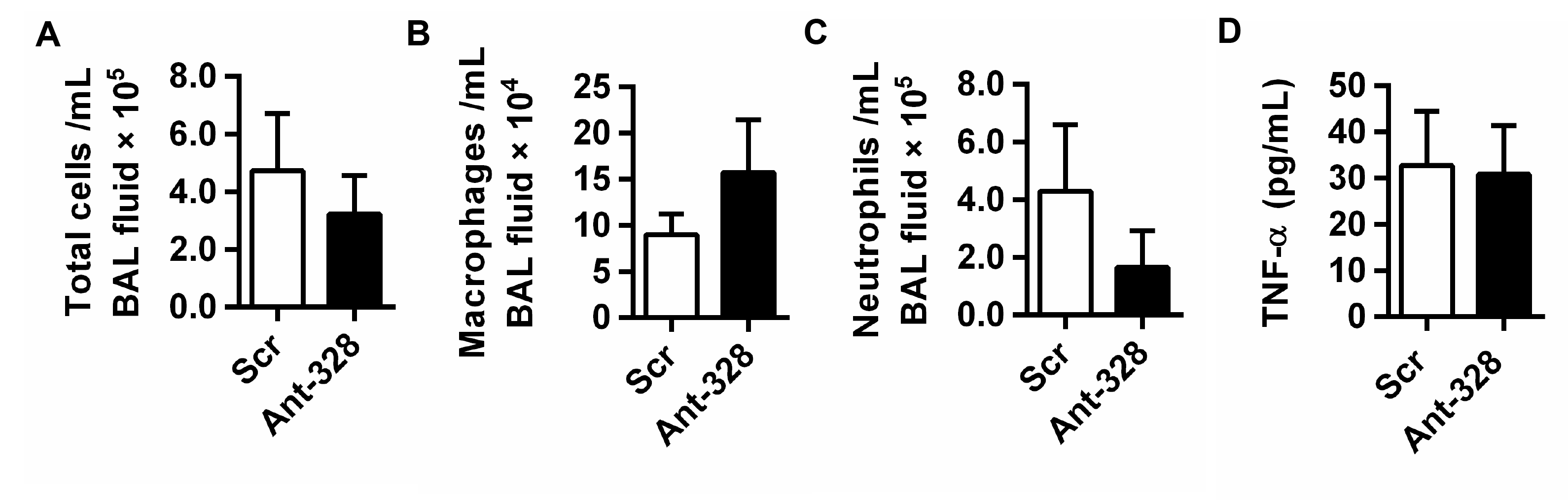

Supplement: S14 Fig — Naïve mice were treated with antagomirs for 12 h. (A) Total cellular infiltrate in BAL fluid was enumerated by cell counts. Cells in BAL fluid were then cytospinned and stained with May-Grunwald. Differential cell counts were performed to identify numbers of (B) macrophages and (C) neutrophils. Lung homogenates were used to determine protein levels of (D) TNF-α by ELISA. Results are expressed as mean ± SEM. (n = 5–6 mice per group) (TIF) [file ppat.1004549.s014.tif]
