# Supplementary material for: Antagonism of miR-328 Increases the Antimicrobial Function of Macrophages and Neutrophils and Rapid Clearance of Non-typeable Haemophilus Influenzae (NTHi) from Infected Lung
Source: PLoS Pathog. 2015 Apr 20;11(4):e1004549. doi: 10.1371/journal.ppat.1004549 (PMC4404141; doi:10.1371/journal.ppat.1004549)
Supplement: S1 Methods — (PDF) [file ppat.1004549.s015.pdf]

Supplemental Methods

NTHi infection & bacterial clearance assays *in vitro*

Macrophages and neutrophils were cultured in DMEM supplemented with 10% heat-inactivated fetal calf serum (FCS) (Sigma-Aldrich), 2mM L-glutamine, 0.1mM sodium pyruvate, and 20mM HEPES. *In vitro* macrophages were infected with MOI 100 NTHi for 8 h while neutrophils were infected with MOI 10 NTHi for 1 h. To measure extracellular bacteria the supernatants were first removed from the macrophage or neutrophil cultures. To measure intracellular bacteria load macrophages or neutrophils were incubated with gentamicin (400µg/mL) for 1 h at 37 ^{o}C to eliminate extracellular bacteria. Cells were then washed three times with PBS before being lysed with 0.25% saponin (Sigma-Aldrich) to release intracellular NTHi. Extracellular and intracellular bacteria were plated onto chocolate agar plates (Oxoid) and incubated overnight at 37 ^{o}C in an atmosphere of 5% CO_2. After 16 h, bacterial colonies were counted.

Measurement of uptake of heat-killed NTHi

Flow cytometry and immunofluorescence microscopy were used to measure the uptake of CFSE labelled heat-killed NTHi. NTHi was labelled with 100µM CFSE (Molecular Probes, before heat-killing) for 10 min at 37°C. Staining was quenched with FCS for 1 min, and bacteria washed, resuspended in PBS, and incubated with cells for 1 h. Cells were harvested for flow cytometry. Numbers of cells positive for CFSE and mean fluorescence intensity were measured using flow cytometry. To visualise the uptake of heat-killed NTHi, macrophages or neutrophils were initially adhered to coverslips. Cells were incubated with rhodamine and DAPI (Sigma-Aldrich) for 5 mins each to stain both cytoplasm and nucleus, respectively. Images were examined with an Olympus BX51 microscope with Olympus 40x objective and images captured using Olympus DP70 camera.

Inhibition of phagocytosis

Primary macrophages isolated from lungs were pretreated with ant-328 or Scr for 12 h before treatment with 2µg/mL Cytochalasin D (Sigma-Aldrich) or vehicle for 1 h. NTHi was labelled with 100µM CFSE (Molecular Probes, before heat-killing) for 10 mins at 37°C. Staining was quenched with FCS for 1 min, and bacteria washed, resuspended in PBS, and incubated with cells for 1 h. Cells were harvested for flow cytometry. Trypan blue (Life Technologies) were used to quench CFSE fluorescence from extracellular bacteria.

miRNA microarray analysis

Mice were challenged with either NTHi or PBS as control and were sacrificed at 24 h post-challenge. Total RNA was extracted from the airways using the Ambion mirVANA kit (Life Technologies) according to the manufacturer's protocol. RNA quality was determined using a bioanalyzer (Agilent). miRNA microarray analysis was performed with mouse miRNA microarray kit release 12.0 (Agilent) according to the manufacturer's protocol. Analysis of microarray data was conducted using Genespring GX software version 11.0.

Isolation of mouse macrophages and neutrophils

Lung tissue from naïve mice was forced through a 70µM cell strainer before being layered on Histopaque 1083 (Sigma-Aldrich) and centrifuged (400 x g, 30min, 20°C). The interface layer containing mononuclear cells was collected and allowed to adhere to a culture plate for 3 h at a concentration of 1 x 10^6 cells before non-adherent cells were removed. Neutrophils were extracted from the bone marrow of naïve mice using a 3 layer Percoll gradient (GE Healthcare) and magnetic bead separation. Briefly, bone marrow cells were isolated from femurs and tibias from both hind legs by flushing the bone with HBSS-EDTA solution. Erythrocytes were lysed with lysis buffer. Cells were then layered on a three-layer Percoll gradient of 78%, 69%, and 52% Percoll and centrifuged (1,500x*g*, 20 min, 20°C) at the slowest deceleration. Cells from the 69%/78% interface were collected and neutrophils further purified by negative immunomagnetic separation using labelling with anti-CD4, -CD8, -CD11c, -CD49b, -CD117, -B220, and -F4/80 purified antibodies (Biolegend). Labelled cells were depleted by magnetic separation by first incubating with BD IMAG streptavidin particles plus-DM (BD Biosciences) according to the manufacturer's protocol. The enriched fraction containing > 96% neutrophils was used.

Adoptive transfer

Macrophages and neutrophils were pre-treated with antagomir miR-328 or scrambled control, or were not exposed, and were harvested and labelled with 5µM of CFSE (Molecular Probes). 5 x 10^5 CFSE labelled macrophages or neutrophils were transferred into the lungs of naïve mice intratracheally in 40µL. 24 h after macrophage instillation and 2 h after neutrophil instillation, mice were challenged with NTHi. Mice adoptively transferred with macrophages were sacrifice 12 h post-inoculation while mice adoptively transferred with neutrophils were sacrificed 6 h post-inoculation.

Cytokine analysis

The concentrations of TNF-α and IL-6 were measured in the supernatants of BAL fluid and lung homogenates, and *in vitro* cell culture supernatants using ELISA kits (eBioscience) according to the manufacturer's instructions.

MAPK phosphorylation analysis

Primary macrophages were treated with MOI 100 NTHi for 1 h. Cell were harvested to analyse p38 and ERK activation by flow cytometry using BD Phosflow™ p38 MAPK and ERK kit (BD Biosciences) according to manufacturer's protocol. Cell lysates were used to measure JNK activation with Human/Mouse/Rat Phospho-JNK Pan specific DuoSet IC ELISA kits (R&D Systems) according to manufacturer's protocol.

Isolation of human monocyte-derived macrohages and neutrophils

Whole blood was obtained from healthy human adult volumteers with written informed consent. PBMCs were isolated by Ficoll centrigugation (GE Healthcare) and cells were adhered to cultures plates for 3 h at 5x10^6 cells/ml after which non-adherent cells were gently

removed. Adherent cells were cultured in 50ng/ml recombinant human M-CSF (Peprotech) to induce macrophage differentiation, and fresh media with M-CSF was replaced on days 3 and 6 of culture. Monocyte-derived macrophages were used on day 7. For neutrophil isolation the remaining Ficoll layer was removed without disturbing the neutrophil/RBC layer. The thin white cell layer of neutrophils above the RBC pellet was collected and resuspended in an equal volume of HBSS and dextran/saline solution (5% dextran T500 in 0.9% NaCl at room temperature), before being incubated in an upright position for 20 min at room temperature. The layer of neutrophils above the sedimented RBC layer was aspirated and the remaining RBCs lysed with lysis buffer. Cells were resuspended in culture media ready for assay.

Synthesis of antagomir oligonucleotides

The complementary antagomirs strands were generated using miRbase and were synthesised by Sigma-Aldrich. miRNAs of interest were inhibited using the antagomirs outlined in table 1

Table 1: Antagomirs design

Antagomir	Sequence
Scrambled antagomir	5'mU.*.mC.*.mA.mC.mA.mA.mC.mC.mU.mC.mC.mU.mA.mG.mA.mA.mA.mG.mA.*.mG.*.mU.*.mA.*.3'-Chol
Antagomir-21	5'mU.*.mC.*.mA.mC.mA.mA.mC.mC.mU.mC.mC.mU.mA.mG.mA.mA.mA.mG.mA.*.mG.*.mU.*.mA.*.3'-Chol
Antagomir-21-3p	5'mU.*.mC.*.mA.mC.mA.mA.mC.mC.mU.mC.mC.mU.mA.mG.mA.mA.mA.mG.mA.*.mG.*.mU.*.mA.*.3'-Chol
Antagomir-146	5'mA.*.mA.*.mC.mC.mC.mA.mT.mG.mG.mA.mA.mT.mT.mC.mA.mG.mT.mT.mC.*.mT.*.mC.*.mA.*. 3'-Chol
Antagomir-376c	5'mU.*.mC.*.mA.mC.mA.mA.mC.mC.mU.mC.mC.mU.mA.mG.mA.mA.mA.mG.mA.*.mG.*.mU.*.mA.*.3'-Chol
Antagomir-223	5’mU.*.mG.*.mG.mG.mG.mU.mA.mU.mU.mU.mG.mA.mC.mA.mA.mA.mC.mU.mG.*.mA.*.mC.*.mA.*. 3’-Chol

“m” represents 2’-OMe-modified phosphoramidites

“*” represents phosphorothioate linkages

“-Chol” was hydroxyprolinol-linked cholesterol to allow permeation of cell membranes

mRNA quantitative polymerase chain reaction

The sequence for the Muc5ac primers were: forward 5’-CGGCCGGAGAAAGTTGGTCCC -3’, reverse 5’-GCACACCCGCCTGGTATGTCC-3’ (Sigma-Aldrich); CD14 primers were: forward 5’-GGAAAGAAACTGAAGCCTTTCTCG-3’, reverse 5’-AACAGCAACAAGCCAAGCACAC-3’ (Sigma-Aldrich); CD36_v2 primers were: forward 5’-GAGATGGCCTTACTTGGGATTGG-3’, reverse 5’-GCCAGTGTATATGTAGGCTCATCCA-3’ (Sigma-Aldrich); CD11b_v2 primers were: forward 5’-AAACTGCTCCTCAAAGCCATTGT-3’, reverse 5’-GGTGACAATCATGTAGATGGCGTA-3’ (Sigma-Aldrich). The housekeeping gene mHPRT was used as a control for the baseline level of cDNA expressed in all samples. The sequence for the mHPRT primers were: forward 5’-AGGCCAGACTTTGTTGGATTTGAA-3’, reverse 5’-CAACTTGCGCTCATCTTAGGCTTT-3’ (Sigma-Aldrich).

miRNA mimic transfection

Primary macrophages were transfected with 50nM of miR-328 mimic or miR-Scr control (Dharmacon) using Lipofectamine RNAiMAX reagent (Life Technologies) according to manufacturer protocol.

Measuring lung function using forced oscillation technique (Flexivent)

Lung function assessment was performed as reported previously [1]. Mice were anaesthesised (i.p.) with 50 μL/10g mixture containing xylazine (2 mg/mL; Troy laboratories, Smithfield, New South Wales, Australia) and ketamine (40 mg/mL; Parnell, Alexandria, New South Wales, Australia). Tracheostomy was performed to insert a cannula into the trachea. Mice were ventilated with a tidal volume of 8 mL/kg at a frequency of 450 breaths/min and a positive end-expiratory pressure of 2 cm H2O. FlexiVent perturbations were performed. Deep inflation, single compartment (snapshot), pressure-volume loops with stepwise or increasing volumes (PVs-V), or pressures (PVs-P), constant phase model (Primewave-8) perturbations were performed. Primewave-8 perturbation was used for tissue elastance and snapshot perturbations were used to measure compliance. Measurements were excluded if the coefficient of determination was lower than 95%.

Reference

1. Li JJ, Wang W, Baines KJ, Bowden NA, Hansbro PM, et al. (2010) IL-27/IFN-gamma induce MyD88-dependent steroid-resistant airway hyperresponsiveness by inhibiting glucocorticoid signaling in macrophages. J Immunol 185: 4401-4409.
